# Copolymers Containing 1-Methyl-2-phenyl-imidazole Moieties as Permanent Dipole Generating Units: Synthesis, Spectroscopic, Electrochemical, and Photovoltaic Properties

**DOI:** 10.3390/molecules27030915

**Published:** 2022-01-28

**Authors:** Irena Kulszewicz-Bajer, Robert Nowakowski, Małgorzata Zagórska, Agnieszka Maranda-Niedbała, Wojciech Mech, Zbigniew Wróbel, Jakub Drapała, Ireneusz Wielgus, Krzysztof P. Korona

**Affiliations:** 1 Faculty of Chemistry, Warsaw University of Technology, Noakowskiego 3, 00-664 Warsaw, Poland; jakub.drapala.dokt@pw.edu.pl (J.D.); iwielgus@ch.pw.edu.pl (I.W.); 2 Institute of Physical Chemistry, Polish Academy of Sciences, Kasprzaka 44/52, 01-224 Warsaw, Poland; rnowakowski@ichf.edu.pl (R.N.); amarandaniedbala@ichf.edu.pl (A.M.-N.); 3 Faculty of Physics, Warsaw University, Pasteura 5, 02-093 Warsaw, Poland; Wojciech.Mech@fuw.edu.pl (W.M.); Krzysztof.Korona@fuw.edu.pl (K.P.K.); 4 Institute of Organic Chemistry, Polish Academy of Sciences, Kasprzaka 44/52, 01-224 Warsaw, Poland; zwrobel@icho.edu.pl

**Keywords:** conjugated donor–acceptor polymers, electrochemical properties, morphological studies, photovoltaic properties

## Abstract

New donor–acceptor conjugated alternating or random copolymers containing 1-methyl-2-phenylbenzimidazole and benzothiadiazole (**P1**), diketopyrrolopyrrole (**P4**), or both acceptors (**P2**) are reported. The specific feature of these copolymers is the presence of a permanent dipole-bearing moiety (1-methyl-2-phenyl imidazole (MPI)) fused with the 1,4-phenylene ring of the polymer main chain. For comparative reasons, polymers of the same main chain but deprived of the MPI group were prepared, namely, **P5** with diketopyrrolopyrrole and **P3** with both acceptors. The presence of the permanent dipole results in an increase of the optical band gap from 1.51 eV in **P3** to 1.57 eV in **P2** and from 1.49 eV in **P5** to 1.55 eV in **P4.** It also has a measurable effect on the ionization potential (IP) and electrochemical band gap (E_g_^CV^), leading to their decrease from 5.00 and 1.83 eV in **P3** to 4.92 and 1.79 eV in **P2** as well as from 5.09 and 1.87 eV in **P5** to 4.94 and 1.81 eV in **P4.** Moreover, the presence of permanent dipole lowers the exciton binding energy (E_b_) from 0.32 eV in **P3** to 0.22 eV in **P2** and from 0.38 eV in **P5** to 0.26 eV in **P4.** These dipole-induced changes in the polymer properties should be beneficial for photovoltaic applications. Bulk heterojunction solar cells fabricated from these polymers (with PC_71_BM acceptor) show low series resistance (r_s_), indicating good electrical transport properties. The measured power conversion efficiency (PCE) of 0.54% is limited by the unfavorable morphology of the active layer.

## 1. Introduction

In the past two decades, significant research efforts have been directed towards the elaboration of conjugated donor–acceptor copolymers of –(D–A)– or –(D–A–D)– types [1,2,3,4,5]. These organic semiconductors frequently exhibit interesting physical and chemical properties that are unmatched or, at least, difficult to match by other groups of organic electroactive compounds. In the majority of cases, they are low band gap semiconductors exhibiting appropriate ionization potential (IP) and electron affinity (EA) values to assure their ambipolarity. As a result, they can serve as components of active layers in various types of ambipolar organic field-effect transistors, characterized by well-balanced n-type and p-type electrical transport [6,7,8,9,10,11,12,13,14,15]. Low ionization potential (IP) and high electron affinity (EA) of D–A and D–A–D polymers, together with the presence of several chromophore groups, promote their reversible electrochemical and spectroelectrochemical behavior [16]. Indeed, these electroactive compounds frequently exhibit several stable oxidation states characterized by colors of distinctly different CIE coordinates, which can be reversibly interconverted by electrochemical switching [17]. Thus, they are very well suited for application as chromophores in electrochemically-active layers of electrochromic devices [18,19,20,21,22]. They are also tested as photodetectors operating in the red and infrared parts of the spectrum [23,24]. Since broad UV–vis–NIR absorption spectra of low band gap –(D–A)– and –(D–A–D)– conjugated polymers usually strongly overlap with the solar spectrum, one of the most promising applications of these compounds is their use as components of active layers in photovoltaic devices, efficiently harvesting solar photons. Recently, spectacular progress in the synthesis of new organic photovoltaic materials has been achieved, resulting in the fabrication of solar cells that exhibit power conversion efficiencies exceeding 18% [25,26]. Photovoltaic processes in polymeric solar cells are complex and are strongly dependent on many factors of different nature, such as the chemical structure of a given macromolecule determining its energy levels and, by consequence, its redox properties, the supramolecular organization of macromolecules in the active layer, the morphology of the layer and others. However, the key step in the conversion of light into electrical energy is the splitting of photo-generated excitons into electron-hole pairs and their subsequent dissociation into free carries. Electrons and holes generated by exciton splitting show a strong coulombic interaction, which can cause significant recombination losses. Thus, the crucial parameters for charges dissociation and separation are low values of exciton-binding energies and high carrier mobilities [27,28]. In contrast to inorganic semiconductors, organic materials exhibit low permittivity values, ε_r_, usually in the range of 3–4 [29]. An increase in active layer permittivity may suppress charge recombination and facilitate carrier transport in the organic layer. A significant increase in the permittivity value can be achieved by increasing electronic polarization [30,31]. Several attempts have been undertaken in this respect, consisting of attaching polarizable side groups or aromatic moieties with permanent dipole moments to the main chains of electroactive-conjugated polymers as well as to non-polymeric components of bulk heterojunction-type active layers such as fullerene derivatives [30,31,32,33]. In a comprehensive paper, Brebels et al. [34] discussed in detail the effect of substituting conjugated polymers or fullerens with groups introducing permanent dipoles. However, the presently available literature data do not bring conclusive evidence for the positive effect of active layer permittivity increase on solar cell performance. Of course, an increase in the permittivity should result in a decrease in exciton-binding energy, facilitating its dissociation into free charges [28,35]. However, other processes may interfere and obscure this effect. It is, therefore, highly desirable to treat these problems in a comprehensive way, i.e., to establish the effect of the permanent dipole presence on redox and spectroscopic properties of the investigated macromolecules as well as on the morphology of the active layer and its photovoltaic performance.

To better identify the effect of the permanent dipole presence on the properties of conjugated polymers, we synthesized two pairs of copolymers consisting of diketopyrrolopyrrole, bithiophene, and *p*-phenylene units. In each pair, the backbones were identical, and the polymers differed only by the presence (or absence) of the dipole introducing moiety, namely, 1-methyl-2-phenyl imidazole. For all polymers, detailed spectroscopic, electrochemical, and morphological studies were undertaken. Bulk-heterojunction-type test devices were fabricated from polymers containing 1-methyl-2-phenyl imidazole groups as donors and fullerene derivatives (PC_71_BM) as acceptors. 

## 2. Results and Discussion

**Scheme 1:**
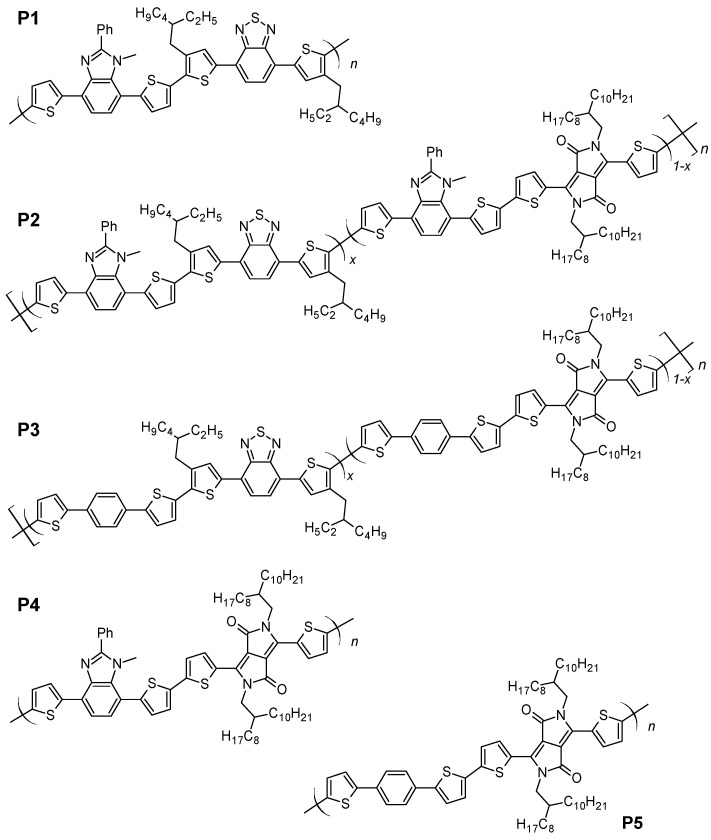
Chemical structures of the studied polymers.

The studied polymers, characterized by the presence of a permanent dipole in their repeat units (**P1**, **P2**, and **P4**) and two reference polymers (**P3** and **P5**), are depicted in Scheme 1. **P1**, **P4,** and **P5** are alternating copolymers, whereas **P2** is a random copolymer of 4,7-dithienobenzimidazole, 4,7-dithienobeznothiadiazole, and 3,6-dithienodiketopyrroloprrole. **P3** is a random copolymer of 1,4-dithienobenzene, 4,7-dithienobeznothiadiazole, and 3,6-dithienodiketopyrroloprrole. Note that the only difference between **P2** and **P3** is the presence of the dipole-bearing moiety, i.e., the 1-methyl-2-phenyl imidazole (MPI) group fused with a phenylene ring in the former. The same applies to **P4** and **P5** containing the diketopyrrolopyrrole (DPP) acceptor. **P1** is different because it contains benzothiadiazole (BT) as an acceptor, whereas in the repeat units of **P2** and **P3,** two acceptor groups can be distinguished, namely, benzothiadiazole (BT) and diketopyrrolopyrrole (DPP).

### 2.1. Synthesis

**Scheme 2:**
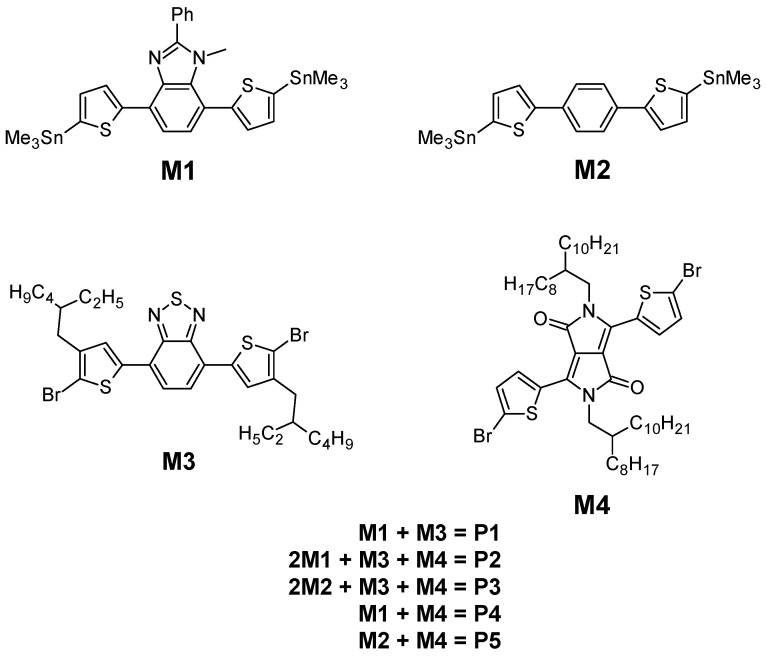
Synthesis of polymers **P1**–**P5**.

The synthetic pathway leading to **P1**–**P5** is presented in Scheme 2. Detailed procedures of the synthesis of the prepared monomers (**M1**–**M4**) (Scheme S1–Scheme S3) and polymers (**P1**–**P5**) can be found in the supplementary data. **M1** was synthesized in a two-step reaction of 1-methyl-2-phenyl-4,7-dibromobenzimidazole,**1** with the thiophene-2-boronic acid pinacol ester (86% yield), followed by lithiation with n-BuLi and subsequent treatment with trimethyltin chloride (98% yield). **M2** was obtained in a similar manner with 90% yield. **M3** and **M4** were synthesized following standard procedures [36]. Polymers were prepared in a microwave reactor via Stille coupling catalyzed with Pd_2_(dba)_3_/P(*o*-tol)_3_. All macromolecular products were soluble in chloroform and chlorobenzene. Molecular masses of fractions soluble in dichloromethane were determined by SEC, indicating M_n_ values in the range from ca. 5.5 × 10^3^ to ca. 1.05 × 10^4^ g/mol and PDI values ranging from 1.5 to 2.0.

### 2.2. Spectroscopic Properties

Donor–acceptor (D-A) polymers containing phenyl imidazole units have been reported in the literature, including those containing methoxystyryl donors [37]. Although interesting from the point of view of their spectroscopic and electrochemical properties, they showed too large an optical band gap (ca. 2.3 eV) and too low electron affinity |EA| (ca. 2.8 eV) to be considered as the components of organic photovoltaic cells. In this paper, we demonstrate that these parameters can be significantly improved in polymers containing phenylimidazole groups by the appropriate selection of the donor and acceptor units in the polymer main chain.

**Figure 1:**
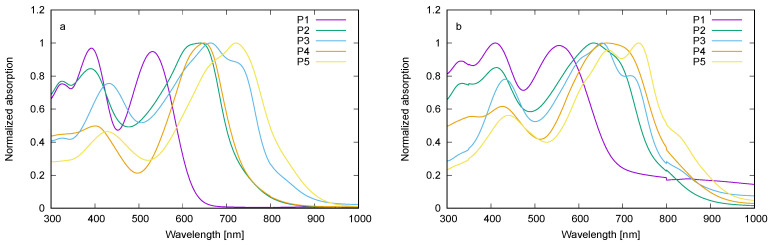
Absorption spectra of the polymers **P1**–**P5**: (**a**) in chloroform solutions, (**b**) in solid-state films.

Absorption spectra of the synthesized polymers strongly depend on the nature of the acceptor units present in the macromolecule. The UV–vis spectrum of **P1** is shown in Figure 1. The position of its least energetic band at λ_max_ = 530 nm in the case of the solution (chloroform) spectrum and at λ_max_ = 560 nm for the spectrum of a thin solid film cast from chloroform were typical of copolymers consisting of thiophene and benzothiadiazole units [38]. Replacement of benzothiadiazole (BT) in **P1** with a significantly stronger acceptor such as diketopyrrolopyrrole (DPP) yields **P4.** The least energetic bands in the solution and solid-state spectra of this polymer are bathochromically shifted by 120 and 98 nm, respectively, compared to the corresponding bands in the spectra of **P1** (see Figure 1 and Table 1). This is not unexpected since stronger donor–acceptor interactions usually result in the significant narrowing of the optical band gap (E_g_^opt^). Incorporation of an additional acceptor to the repeat unit is usually beneficial for photovoltaic applications because it may result in a significant broadening of the absorption bands, leading to their better overlapping with the solar spectrum [39]. This broadening, together with a significant bathochromic shift of the absorption band, is very pronounced in the case of **P2**, which contains two acceptor groups in its repeat unit (**BT** and **DPP**). It is also clearly observed for **P3**. **P4,** and **P5,** which do not contain the benzothadiazole chromophore in their repeat units. Their absorption bands are narrower and bathochromically shifted with respect to the corresponding bands of **P2** and **P3**. The same trend is observed in the solid-state spectra recorded for thin films cast from chloroform (see Figure 1 and Table 1). Moreover, the absorption bands in the solid-state spectra of the reference polymers (**P3** and **P5**) are structured, showing clear maxima at 653 and 720 nm for **P3** and a shoulder at 850 nm, accompanied by two clear maxima at 672 and 738 nm in the case of **P5**. The presence of these bands can be tentatively attributed to the formation of semi-crystalline phases of these polymers, as was suggested by Li et al. [40] for DPP polymers with alkyl side chains.

It should be noted that the least energetic bands in the solution and solid-state spectra of the reference polymers (**P3** and **P5**) are bathochromically shifted with respect to the corresponding bands of the permanent dipole-bearing polymers (**P2** and **P4**), indicating better conjugation in the former (see Figure 1 and Table 1). Molar absorption coefficients of **P2** and **P3** are higher than those determined for **P1**, P**4,** and **P5**. It should, however, be stressed that they contain more chromophore groups in their repeat units. For the same reason, the molar absorption coefficient of **P2** is higher than that determined for **P3** since in the repeat unit of the former, an extra chromophore (1-methyl-2-phenyl benzimidazole) is present. 

Optical band gaps, E_g_^opt^, determined from the absorption onset in the spectra of thin films, are listed in Table 1. As expected, the largest band gap, 1.84 eV, is observed for **P1**, i.e., the only polymer containing a weaker acceptor (BT) instead of a stronger DPP one. Optical band gaps of **P2** and **P4** are similar (1.57 and 1.55 eV) and consistently larger by 60 meV compared to the gaps of the reference polymers, i.e., **P3** and **P5**. The E_g_^opt^ of **P5** is slightly lower than the band gap of a very similar polymer reported by Li et al. [41], which differs from **P5** by shorter alkyl substituents attached to the DPP group. 

**Figure 2:**
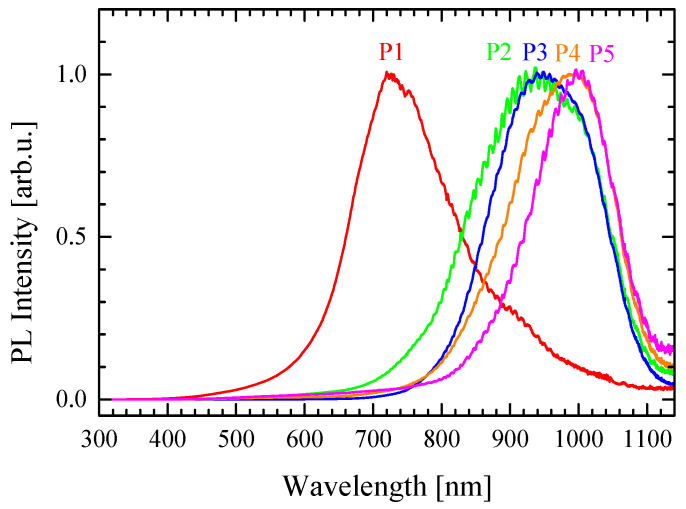
Photoluminescence spectrum of thin films of **P1**–**P5**.

Thin films of **P1** show weak photoluminescence with a maximum at ca. 720 nm (1.72 eV). **P2** and **P3,** as well as **P4** and **P5,** emit at the same λ_max._ of 942 nm (1.32 eV) and 998 nm (1.24 eV), respectively. In all cases, the Stokes shifts are rather large, exceeding 150 nm. Note that the emission bands of the polymers bearing the permanent dipole (**P2** and **P4**) are broadened on their higher energy side (see Figure 2).

### 2.3. Electrochemical Properties

**Figure 3:**
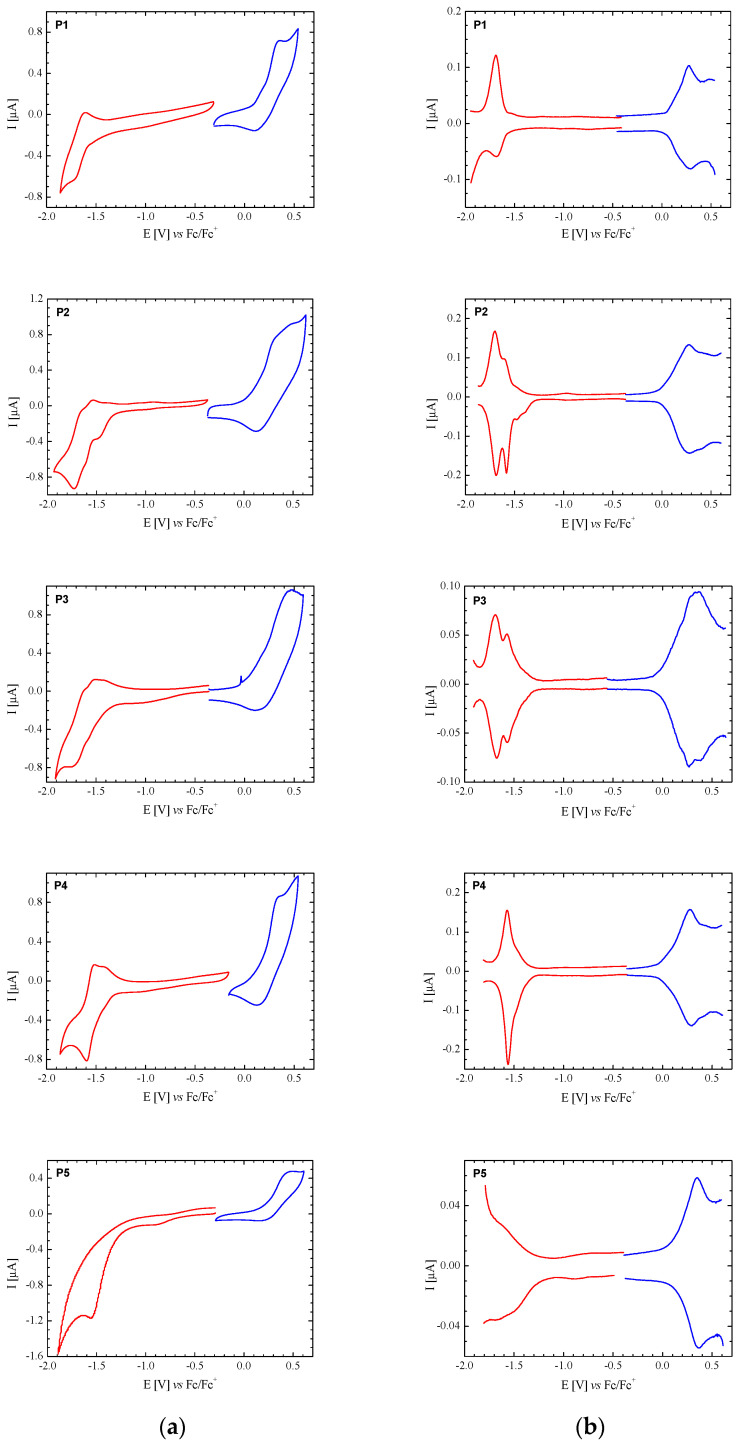
Cyclic voltammograms (**a**) and differential pulse voltammograms (**b**) registered for the polymers **P1**–**P5** dissolved in 0.1 M Bu_4_NBF_4_/dichloromethane electrolyte. CV: scan rate = 50 mV/s and DPV: modulation time = 50 ms, modulation amplitude = 10 mV, step potential = 5 mV.

Redox properties of conjugated polymers are of crucial importance for their electrochemical and electronic applications. For these reasons, cyclic voltammetry (CV) and differential pulse voltammetry (DPV) are routinely used for the characterization of new conjugated polymers and for the determination of their ionization potential (IP) and electron affinity (EA). In Figure 3, cyclic voltammograms of **P1**–**P5** are presented, whereas the determined redox potentials are collected in Table 2.

There are some common features in the electrochemical properties of all studied polymers. They all undergo one-step, partially reversible oxidation at relatively low potentials. The reduction of **P1**–**P4** is also partially reversible, **P5** being the only exception whose reduction is purely irreversible. **P1** undergoes oxidation at the same potential as **P4**, but its reduction potential is lower by 120 mV compared to that of **P4**. Since both polymers differ by the acceptor unit nature only, the more difficult reduction of **P1** reflects the weaker electroaccepting properties of benzothiadiazole compared to diketopyrrolopyrrole. **P2** and **P3** contain both (BT and DPP) acceptor units and, for this reason, undergo a two-step reduction. By comparison with the data obtained for **P1**, **P4**, and **P5**, it can be concluded that the cathodic peak at higher potentials corresponds to the reduction of DPP, whereas that at lower potentials corresponds to the reduction of BT. The presence of the permanent dipole in **P2** and **P4** meaningfully affects their redox properties, decreasing their oxidation potential compared to **P3** and **P5** and increasing the reduction potential in the case of **P4** (see Table 2).

From the oxidation and reduction potentials of the studied polymers, it is possible to calculate their ionization potentials (IPs) and electron affinities (EAs), provided that the first oxidation process corresponds to the oxidation of the repeat unit to a radical cation, whereas the first reduction process leads to its transformation into a radical anion. It should be, however, noted that the electrochemically determined IPs and EAs comprise several contributions that may alter their values. These are, for example, interactions of the ionized macromolecules with their environment, which depend on their chemical nature, polarization, and solvation energy. Thus, an important question arises of whether IP and EA values derived from electrochemical data properly are correlated with those obtained by direct methods, i.e., ultraviolet photoelectron spectroscopy (UPS) and inversed photoelectron spectroscopy (IPES). Sworakowski et al. [42,43,44], based on numerous experimental electrochemical and spectroscopic data published in the literature, found a clear correlation between electrochemical and photoelectron spectroscopy data. The data collected in Table 2 show that the presence of the permanent dipole lowers IP since its values determined for **P2** and **P4** are lower than the corresponding IP values found for **P3** and **P5**. EA values are less affected by the presence of the permanent dipole but the lowering of their values in **P2** and **P4** is measurable. 

Assuming the validity of Koopmans theorem (E_HOMO_ = −IP, E_LUMO_ = EA), it is possible to calculate the so-called “electrochemical band gap” (E_g_^CV^). These gaps are very similar for **P3** and **P5,** being, however, slightly narrower for **P2** and **P4**. E_g_^CV^ values are consistently larger than optical E_g_^opt.^ for all studied polymers (**P1**–**P5**). The difference between the electrochemical and the optical band gaps can be treated as an effective singlet exciton-binding energy E_b_ [35]: E_b_ = E_g_^CV^ − E_g_^opt^. Willems et al. estimated E_b_ for different polymers containing DPP units and found that E_b_ is equal to ca. 0.44 eV, independent of the specific polymer [45]. This value was clearly higher than that determined for **P5** (0.38 eV, see Table 2), i.e., the polymer in which DPP was the sole acceptor unit. By comparing **P2** with **P3** and **P4** with **P5**, it becomes clear that the presence of permanent dipole-bearing units in the polymer chain lowers the exciton binding energy by 100–120 mV. Thus, the obtained results clearly indicate that the local dipoles should play a beneficial role in the charge generation associated with photovoltaic phenomena.

### 2.4. Thin Films Morphology

Intuitively, it seems clear that the presence of permanent dipoles in the macromolecules of conjugated polymers should significantly modify their capability of aggregation, and, by consequence, it should lead to different supramolecular organizations and morphologies of the resulting solution cast films. This is an important aspect of conjugated polymers solution processing, especially in view of their photovoltaic applications, since morphology is one of the dominant factors influencing electrical transport properties in organic electronic devices, including photovoltaic cells. Thus, one of the main problems to be discussed in this research is the effect of fusion of the permanent dipole-bearing groups (methyl-2-phenyl imidazole, MPI) with the polymer main chain on the morphology of the resulting polymers. Hence, detailed comparative AFM observations of monolayers of **P4** and monolayers of its dipole-free analog, i.e., **P5** were performed. 

**Figure 4:**
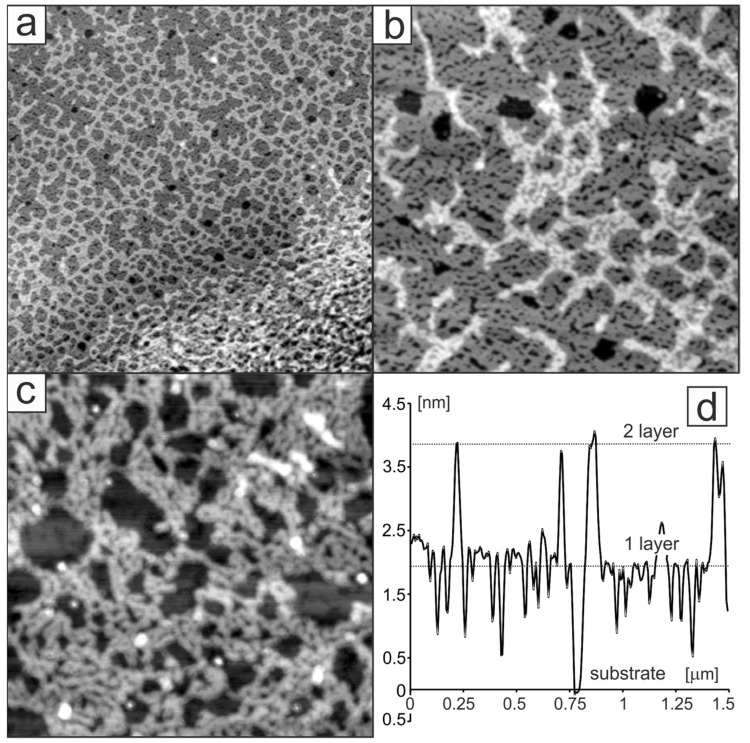
AFM images (**a**–**c**) and the corresponding line profile (**d**) of mono-, bi-, and multilayers of **P5** deposited on mica from its solutions in chlorobenzene. Scanning area: (**a**) multilayer 5 × 5 μm^2^, (**b**) bilayer 1.5 × 1.5 μm^2^, (**c**) monolayer 1 × 1 μm^2^, (**d**) surface profile of selected line from image (**b**).

Figure 4 shows the AFM images (a–c) and the corresponding line-profile (d) of the reference polymer (**P5**) layers deposited directly from its solution in chlorobenzene (optimal solvent for the tested group of polymers). A representative image of the larger area (5 µm × 5 µm) is presented in the image in Figure 4a. As a consequence of the polymer concentration gradient, a transition is clearly observed: from a monolayer (in the upper-left corner of the image) through to an incomplete bilayer (dominant in the middle part of the image) to three-layer coverage (bottom-right corner). Subsequent higher resolution images provide more details. The bilayer surface is presented in Figure 4b. As expected, three levels can be easily distinguished in this case: small areas of the uncovered substrate (darker parts), macromolecules in the monolayer (grey areas dominating the image), and locally observed individual molecules or their small aggregates in the second layer (bright areas of elongated shape). Additionally, the corresponding profile of the selected line is presented in Figure 4d, which confirms the same thickness of the first and the second layer (ca. 2 nm). The AFM image of the area where the monolayer strongly dominates is shown in Figure 4c. The above observations demonstrate the tendency of **P5** to form films of layered structures, characterized by a small distance between the chains of adjacent molecules in each layer. This is evidenced by clearly visible areas of densely packed polymers in the monolayer as well as in the bilayer. These morphological features clearly indicate that multilayer films of **P5,** showing a compact structure, can be deposited in these conditions.

**Figure 5:**
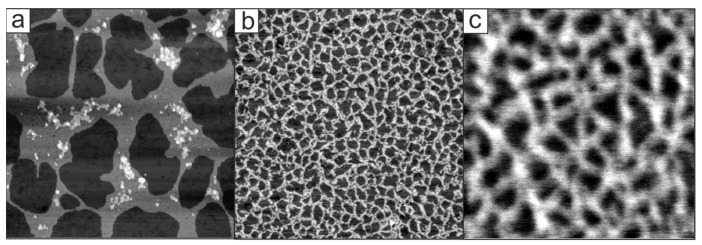
AFM images of (**a**) **P5**, (**b**) and (**c**) **P4** layers deposited on mica from solutions in chloroform. Scanning area: (**a**,**b**) 2.5 × 2.5 μm^2^, (**c**) 1 × 1 μm^2^.

As already stated, the main chain of **P4** is the same as that of **P5**. The only difference between these polymers is the presence of the permanent dipole-bearing group (1-methyl-2-phenyl imidazole) fused with the phenylene ring of the main chain in the case of **P4**. The presence of this dipole results in the distinctly different supramolecular aggregation patterns of this polymer. The first dissimilarity concerns the selection of the solvent for thin layer deposition. In the case of **P4**, chloroform has to be used for casting. It is, therefore, tempting to compare the topologies of the monolayers of **P4** and **P5**, deposited from chloroform, in the same conditions. Chloroform is more polar than chlorobenzene, and the effect of this higher polarity additionally increases the effect of intermolecular interactions in the case of **P5**. As a consequence, the monolayer of **P5**, deposited from this solvent, is characterized by randomly distributed larger areas of very tightly packed macromolecules (Figure 5a). More polar **P4**, on the other hand, forms, in the same conditions, a homogeneous network of loosely packed macromolecules (Figure 5b,c). The resulting mesh is characterized by much larger distances between adjacent molecules (approx. 110–160 nm). 

Summing up this part of the research, significant differences in the supramolecular organization of both polymers should be emphasized. The **P4** polymer forms layers with much weaker packing compared to reference polymer **P5**. This is a direct consequence of electrostatic interaction between the polar MPI groups presented in this macromolecule. This may have a negative impact on the electrical properties of the layers it forms.

### 2.5. Photovoltaic Properties

Before discussing in detail the photovoltaic properties of the newly developed polymers containing 1-methyl-phenyl imidazole, it is instructive to justify their application as donors in organic cells, together with PC_71_BM acceptor. As already mentioned, by selecting appropriate electron-rich and electron-poor units in the polymer main chain, we could adjust the crucial material parameters of the donor component of the cell. These are ionization potential (IP) and electron affinity (EA). IP values of **P1**–**P5** are in the range of 4.92 to 5.09 eV, whereas the |EA| values vary from 2.99 to 3.22 eV. IP and |EA| of PC_71_BM are equal to 5.87 and 3.91 eV, respectively [46]. It is generally accepted that in properly operating photovoltaic cells, the difference between the values of |EA| of the donor and |EA| of the acceptor should be superior to 0.3 eV if excitons are generated in the donor phase. By symmetry, the difference between IP of the donor and IP of the acceptor should also be superior to 0.3 eV if excitons are formed in the acceptor phase [47,48,49]. Nanocomposites of **P1**–**P5** with PC_71_BM excessively fulfill these conditions. Moreover, as shown in Section 2.3, the presence of a permanent dipole in **P1**, **P2**, and **P4** lowers the exciton-binding energy by ca. 30%, facilitating, in this manner, the generation of free charge carriers. Finally, our investigations of the luminescent properties of blends of PC_71_BM with the synthesized polymers showed the effective quenching of the luminescence in this two-phase system (see Figure S6 in the supplementary information). Thus, by estimating the above-discussed thermodynamic and spectroscopic properties of **P1**, **P2**, and **P3**, it quickly became clear that they should have been considered promising candidates for application in organic photovoltaic cells. **P2** and **P4** were selected for these investigations because of their better matching of the solar spectrum.

**Figure 6:**
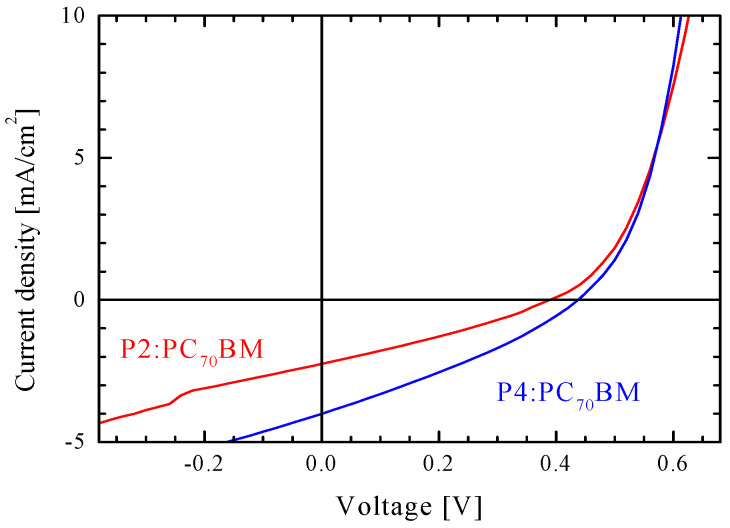
Current—voltage characteristics of solar cells with **P2**:PC_71_BM (red) and **P4**:PC_71_BM (blue) heterojunctions.

The photovoltaic properties of **P2** and **P4** were tested in the bulk heterojunction-type solar cells of the following architecture: ITO/PEDOT:PSS/polymer:PC_71_BM/InAl. The blends were solution-processed from chloroform. Under standard 1.5 AM illumination (100 mW/cm^2^), the investigated solar cells generated current density in the range of a few mA/cm^2^ (see Figure 6). **P4** turned out to be a more effective component of the bulk heterojunction since **P4**-based cells were ca. twice more efficient than the **P2**-based ones. The absolute value of current density |*J*| decreased with increasing forward voltage, indicating high leakage. The characteristics were analyzed, assuming the parasitic resistance model (series resistance and shunt resistance). The data of electrical parameters of **P2**:PC_71_BM and **P4**:PC_71_BM based solar cells are presented in Table 3. The cells had 8 pixels each; thus, the presented data correspond to average values, and their uncertainties are given. 

The measured low *r*_S_ values (3–6 Ω⋅cm^2^) suggest the good mobility of charge carriers. It should be, however, noted that both devices show rather low values of short circuit current. The photocurrent measured for the blends with **P4** is ca. twice higher than that for the blends with **P2**, but it is ca. three times lower than the value reported by Li et al. [40] for a polymer very similar to **P5** (analog of P5 without the permanent dipole) or for PTB7-based (poly[4,8-bis(5-(2-ethylhexyl)thiophen-2-yl)benzo[1,2-b;4,5-b’]dithiophene-2,6-diyl-alt-(4-(2-ethylhexyl)-3-fluorothieno[3,4-b]thiophene-)-2-carboxylate-2-6-diyl)]) of similar architecture [50]. The absorption spectra of **P2** and **P4**, although different, cover a very similar spectral range; thus, the measured difference of the photocurrent cannot be related to the better harvesting of the solar flux and the greater number of excitons formed in the blend with **P4** compared to that fabricated with **P2**. Moreover, the IP and EA values (which correspond to HOMO and LUMO levels) for **P2** and **P4** are similar. Low values of the measured photocurrent in both cases should, therefore, originate from weak light absorption of the blends and high leakage of the layer (low *r_sh_* value). This leakage can be caused by the poor morphology of the layer. Monolayers of neat polymers form a kind of mesh with a large distance (above 100 nm) between adjacent aggregates (see Figure 5), and this tendency is maintained in thicker films and in the blends with fullerene derivatives.

The open-circuit voltage (*V_oc_*) of the fabricated cells was lower than expected, considering the values of the HOMO and LUMO energies, and lower than predicted from the relation between the open-circuit voltage and the oxidation potential, as proposed by Willems et al. [45]. It can be supposed that the presence of the permanent dipoles in the macromolecules of **P2** and **P4** may provoke energetic disorder. Different local positions of these dipoles may cause a broad distribution of the electronic density of states, which could be more pronounced at the donor–acceptor interfaces. Thus, *V_oc_* losses can be related to the distribution of electronic states and the presence of interfacial energetic traps. The distribution of electronic states and energetic traps is dependent on the blend morphology. Thus, the morphological disorder may affect the material properties, leading to low values of the measured electrical parameters.

**Figure 7:**
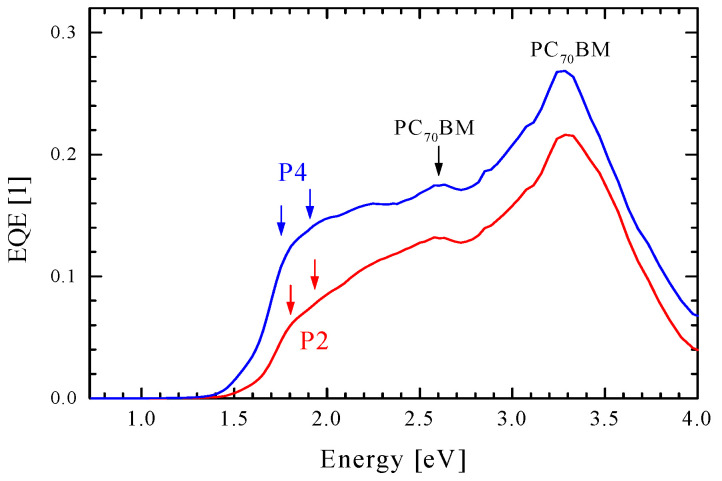
Quantum external efficiency (EQE) spectra of solar cells with **P2**:PC_71_BM (red) and **P4**:PC_71_BM (blue) heterojunctions. Energies of absorption peaks of **P2**, **P4**, and PC_71_BM are marked by arrows.

External quantum efficiency (EQE) spectra were obtained by measuring the photocurrent in the short circuit mode (I_SC_). The EQE was of the order of 20%, which means that one out of five photons was successfully transformed into a charge carrier, reaching the cathode. Since the absorbing layers were relatively thin (ca. 100 nm), only half of the photons were absorbed and about half of the photoexcited electrons arrived at the cathode. The cells with **P4** were about two times more efficient than those with **P2**. As indicated in Figure 7, absorption bands of the donor and acceptor components of the bulk heterojunctions (marked by arrows) are located in the visible part of the spectrum (for the shape of the spectra of **P2** and **P4**, see Figure 1; the spectrum of PC_71_BM can be found in [50]). This means that both components can efficiently absorb photons, and the interface charge transfer can proceed in both directions. The absorption efficiencies of **P2** and **P4** were lower than that determined for PC_71_BM. Different spectral ranges of measurements should, however, be taken into consideration. In the 2 eV range, significantly more solar photons are expected than in the 3 eV range, so the former is more important for the power conversion efficiency (PCE).

Taking into account the IP and |EA| values of **P2** and **P4** and their absorption spectra matching the solar spectrum, reduced exciton dissociation energy due to the presence of permanent dipoles, and efficient photoluminescence quenching in the presence of PC_71_BM, much better cell parameters should be expected. Morphological studies of the active layers clearly showed that unfavorable morphology was the main reason for the cell parameters worsening. Modifications of the processing conditions were, therefore, undertaken. In particular, BHJ layers of **P2** and **P4** with PC_71_BM were processed from different solvents (chloroform or chlorobenzene) using different additives such as diiodobenzene (DIO) and o-dichlorobenzene (DCB). Although for individual cells, higher PCE values were obtained, the average values calculated for the set of eight cells did not improve (see Table S1 in the supplementary information). Again, unfavorable film morphology was the main reason for this lack of cell parameter improvement (see Figures S8, S9, S11, and S12). It is clear that further improvement of the cell parameters requires the elaboration of processing methods, leading to better BHJ morphologies. Such investigations are in progress.

## 3. Materials and Methods

Detailed procedures of the synthesis of the prepared monomers (**M1**–**M4**) and polymers (**P1**–**P5**) can be found in the supplementary materials. This involves:

(i) description of all reagents and procedures used in the preparation of the monomers and the studied polymers together with spectroscopic (NMR, IR) data of intermediate and final products as well as their elemental analyses;
(ii) absorption spectra of the studied polymers in chlorobenzene solutions and in solid-state films prepared from chlorobenzene solutions;
(iii) description of cyclic voltammetry (CV) and differential pulse voltammetry (DPV) experiments;
(iv) description of AFM measurements;
(v) device preparation and characterization;
(vi) morphology of the active layer investigated with an optical microscope using Nomarsky contrast.

## 4. Conclusions

To summarize, we have synthesized new donor–acceptor-type conjugated copolymers modified by the fusion of a permanent dipole-bearing moiety (1-methyl-2-phenyl imidazole, MPI) to the 1,4-phenylene group of the main chain. For comparative reasons, we prepared periodic copolymers of the same main chain but deprived of the MPI groups. The presence of the permanent dipole increases the optical band gap of the studied polymers but lowers their ionization potential (IP) and their electrochemical band gap. Moreover, their exciton binding energy is also reduced, as derived from a combination of spectroscopic and electrochemical investigations. These beneficial, dipole-induced changes of the polymers’ properties, combined with appropriate IP and |EA| and good spectral matching with the solar spectrum, are not reflected in the parameters of the tested bulk heterojunction-type solar cells. The measured PCE values are strongly limited by the unfavorable morphology of the active layers.

## Figures and Tables

**Table 1:** Molecular masses, maxima of absorption peaks, molar absorption coefficients, and optical band gaps, E_g_^opt^, of **P1**–**P5**.

Polymer	M_n_ [kg/mol]	M_w_ [kg/mol]	PDI	λ_peak_^s (a)^ [nm]	λ_peak_^f (a)^ [nm]	λ_onset_^f^ [nm]	E_g_^opt. (b)^ [eV]	ε⋅10^−4 (c)^ [M^−1^cm^−1^]
**P1**	5.5	8.4	1.53	530	560	672	1.84	5.56
**P2**	5.5	8.2	1.49	620 640	635 sh 690	790	1.57	12.15
**P3**	6.6	13.4	2.01	655 sh 730	653 720	820	1.51	8.15
**P4**	10.6	20.8	1.96	650	658	800	1.55	6.05
**P5**	7.9	10.9	1.38	720 sh 660	672 738 sh 850	832	1.49	6.58

^(a)^ λ_peak_^s^ in CHCl_3_ solution; λ_peak_^f^ and λ_onset_^f^ for films prepared from CHCl_3_ solution. ^(b)^ E_g_^opt.^ determined from the onset of the absorption band. ^(c)^ molar absorption coefficient, ε.

**Table 2:** Electrochemical properties of polymers **P1**–**P5**. Data presented: oxidation and reduction potentials vs. Fc/Fc^+^; ionization potential, IP; electron affinity, EA; electrochemical band gap, E_g_^CV^; exciton binding energy, E_b_.

Polymer	E_ox_ ^(a)^ [V]	E_ox_^onset (b)^ [V]	E_red1_ ^(a)^ [V]	E_red2_ ^(a)^ [V]	E_red_^onset (b)^ [V]	IP ^(c)^ [eV]	EA ^(c)^ [eV]	E_g_^CV^ [eV]	E_b_ [eV]
**P1**	0.28	0.21	−1.69	−	−1.56	5.03	2.99	2.04	0.20
**P2**	0.28	0.11	−1.59	−1.70	−1.44	4.92	3.13	1.79	0.22
**P3**	0.32	0.18	−1.56	−1.69	−1.41	5.00	3.17	1.83	0.32
**P4**	0.28	0.13	−1.57	−	−1.44	4.94	3.13	1.81	0.26
**P5** ^(d)^	0.33	0.26	–1.65	–	–1.37	5.09	3.22	1.87	0.38

^(a)^ peak potentials determined from DPV, the potential values given vs. Fc/Fc^+^. ^(b)^ E^onset^ were determined from CV, the potential values given vs. Fc/Fc^+^. ^(c)^ IP = |e|⋅(1.15·E_ox_^onset^ + 4.79) eV, EA = e·(1.18·E_red_^onset^ + 4.83) eV, according to [42,43,44]. ^(d)^ low-molecular weight fraction.

**Table 3:** Photoelectric parameters of the investigated solar cells.

Sample		*V_oc_* [V]	*J_sc_* [mA/cm^2^]	*r_sh_* [Ω cm^2^]	*r_S_* [Ω cm^2^]	FF	PCE %
**P2**:PC_71_BM	<x>	0.39	2.3	218	6	0.296	0.26
2:3	±σ	0.02	0.2	20	1	0.005	0.02
**P4**:PC_71_BM	<x>	0.44	4.0	147	2.8	0.303	0.54
2:3	±σ	0.02	0.6	17	0.2	0.004	0.09

*V_oc_*—open-circuit voltage, *J_sc_*—short circuit current, *r_sh_* and *r_S_*—shunt and series resistivities, FF—fill factor, PCE—power conversion efficiency.

## Data Availability

The data presented in this study are available from the supplementary materials.

## Supplementary Materials

The following are available online: the detailed synthesis of the monomers and polymers, Scheme S1: Synthesis of monomer **M1**, Scheme S2: Synthesis of monomer **M2**, Scheme S3: Syn-thesis of monomer **M3**, Figures S1–S5: FTIR spectra of the polymer **P1**–**P5**, respectively, Figure S6: Absorption spectra of the polymers **P1**–**P5**: (a) in chlorobenzene solutions, (b) in solid-state films; Figure S7: Photoluminescence spectra of neat films of **P1** and **P2** and their blends with PC_71_BM (1:2), description of electrochemical and AFM measurements, Figure S8: AFM image of **P2** layers deposited on mica from solution in chlorobenzene, Figure S9: AFM image of **P4** layers deposited on mica from solution in chlorobenzene, fabrication of photovoltaic cells, Figure S10: Photograph of solar cell with **P2**:PCBM heterojunction, Figure S11: Solar cell **P2**:PC_71_BM—a lot of small bubbles are visible caused by evaporation of the solvent. The bubbles reduce the working area, Figure S12: Solar cell **P4**:PC_71_BM—the bubbles are bigger than in the case of P2 but the flat area between them is greater than in the case of **P2**.

## Sample Availability

Samples of the compounds are not available from the authors.
