# Supplementary material for: Copolymers Containing 1-Methyl-2-phenyl-imidazole Moieties as Permanent Dipole Generating Units: Synthesis, Spectroscopic, Electrochemical, and Photovoltaic Properties
Source: Molecules. 2022 Jan 28;27(3):915. doi: 10.3390/molecules27030915 (PMC8840365; doi:10.3390/molecules27030915)
Supplement: Supplementary file 1 [file molecules-27-00915-s001.zip › molecules-1537596-supplementary.pdf]

Supplementary Materials

Copolymers Containing 1-Methyl-2-phenyl-Imidazole Moieties as Permanent Dipole Generating Units: Synthesis, Spectroscopic, Electrochemical and Photovoltaic Properties

I. Kulszewicz-Bajer [1]*, R. Nowakowski [2], M. Zagórska [1]*, A. Maranda-Niedbała [2], W. Mech [3], Z. Wróbel [4], J. Drapała [1], I. Wielgus [1], K.P. Korona [3]

[1] Faculty of Chemistry, Warsaw University of Technology, Noakowskiego 3, 00-664 Warsaw, Poland

[2] Institute of Physical Chemistry, Polish Academy of Sciences, Kasprzaka 44/52, 01-224 Warsaw, Poland

[3] Faculty of Physics, University of Warsaw, Pasteura 5, 02-093 Warsaw, Poland

[4] Institute of Organic Chemistry, Polish Academy of Sciences, Kasprzaka 44/52, 01-224 Warsaw, Poland

Index

Synthesis 2

Optical studies 10

Electrochemical studies 11

Morphological studies – AFM measurements 12

Device fabrication and characterization 12

Morphology of the active layer 14

Bibliography 15

Synthesis

Characterization techniques

^{1}H and ^{13}C NMR spectra were recorded on a Varian Mercury (500 and 125 MHz) spectrometer and referenced with respect to TMS and solvents. IR spectra were monitored on Bio-RAD FTS-165 spectrometer using KBr pellets. UV-Vis-NIR spectra were registered using a Cary 5000 (Varian) spectrometer. Mass spectra were measured by EI method on an AMD 604 mass spectrometer. Gel permeation chromatography (GPC) was performed on LAB Alliance equipped with a column Jordi Gel DVB Mixed Bed using dichloromethane as a solvent and polystyrene standards. All synthesized compounds studied were subject to C, H, N elemental combustion analysis.

Reagents

1,4-diiodobenzene, 4,7-dibromobenzothiadiazole, 3,6-di(thiophen-2-yl)pyrrolo[3,4-c]pyrrole-1,4(2H,5H)-dione, thiophene-2-boronic acid pinacol ester, trimethyltin chloride (1 M solution in hexane), n-butyllithium (2.5 M in hexane), benzyltriethylammonium chloride, BTENCl, tetrakis(triphenylphosphine) palladium(0), $Pd(PPh_3)_4$, tris(dibenzylideneacetone) dipalladium(0), Pd_2dba_3, *orto*-tolylphosphine, NBS, Na_2CO_3, anhydrous THF, chlorobenzene were purchased from Aldrich.

All glassware was oven dried, assembled hot, and cooled under a dry argon stream before use. All reactions were performed under dry argon.

1-methyl-2-phenyl-4,7-dibromobenzimidazole, **1** was prepared according to the procedure described in [1]. The compounds **4**–**M3**, **7** and **M4** were prepared according to the procedures described in [2].

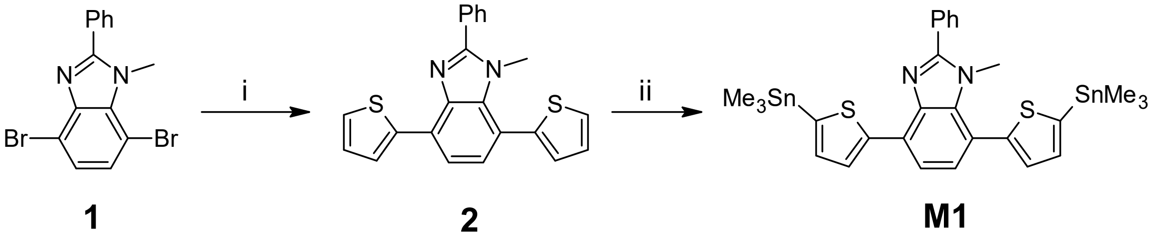

Scheme S1. Synthesis of monomer **M1**: i) thiophene-2-boronic acid pinacol ester, $Pd(Ph_3P)_4$, toluene/H_2O, Na_2CO_3, 100 °C, ii) n-BuLi, –78 °C, Me_3SnCl –78 °C to RT.

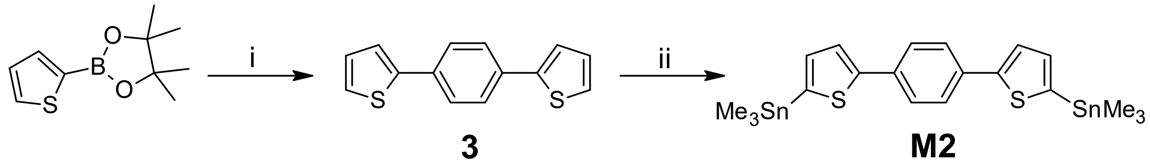

Scheme S2. Synthesis of monomer **M2**: i) 1,4-diiodobenzene, $Pd(Ph_3P)_4$, toluene/H_2O, Na_2CO_3, 100 °C, ii) n-BuLi, –78 °C, Me_3SnCl –78 °C to RT.

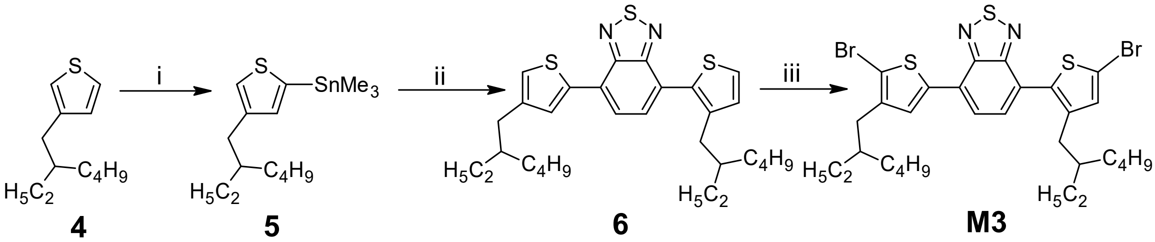

Scheme S3. Synthesis of monomer **M3**: i) n-BuLi, TMEDA, ether, RT, Me_3SnCl, RT, ii) 4,7-dibromobenzothiadiazole, $PdCl_2(Ph_3P)_2$, THF, iii) NBS, THF, DMF.

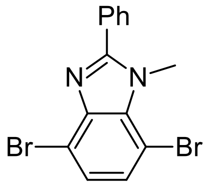

1-methyl-2-phenyl-4,7-dibromobenzimidazole, 1

^{1}H NMR (500 MHz, $CDCl_3$), δ, 7.72–7.70 (m, 2H), 7.54–7.52 (m, 3H), 7.33 (d, J = 8.5 Hz, 1H), 7.30 (d, J = 8.5 Hz, 1H), 4.10 (s, 3H).

^{13}C NMR (125 MHz, $CDCl_3$) δ, 156.50, 143.55, 133.86, 130.42, 130.15, 129.49, 128.85, 128.24, 126.49, 113.17, 102.64, 34.98.

EI m/z = 364.9, calculated for $C_{14}H_{10}N_2Br_2$: 365.05

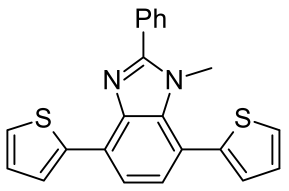

1-Methyl-2-phenyl-4,7-bis(thiophene)benzimidazole, 2

To a three-neck flask under Ar was added 1-methyl-2-phenyl-4,7-dibromobenzimidazole, **1** (1.83 g, 5 mmol), thiophene-2-boronic acid pinacol ester (2.31 g, 11 mmol), BTENCl (0.06 g), tetrakis(triphenylphosphine) palladium(0) (144.5 mg, 0.125 mmol), toluene 15 ml and 15 ml of degassed 2 M water solution of Na_2CO_3. The mixture was stirred and heated at 100 °C under Ar for 24 h. After cooling the solution was poured into 50 ml of water and extracted with ethyl acetated. The organic phase was dried over Na_2SO_4. The crude product was purified by gel column chromatography eluting with dichloromethane : hexanes, 2:1 to give the product as a white solid (1.6 g, 4.3 mmol) with 86% yield.

^{1}H NMR (500 MHz, $CDCl_3$) δ, 8.22 (dd, J = 3.5, 1.0 Hz, 1 H), 7.84–7.82 (m, 2H), 7.60 (d, J = 7.5 Hz, 1 H), 7.53–7.50 (m, 3H), 7.42 (dd, J = 5.0, 1.0 Hz, 1H), 7.37 (dd, J = 5.0, 1.0 Hz, 1H), 7.28 (d, J = 7.5 Hz, 1 H), 7.19–7.14 (m, 3H), 3.53 (s, 3H).

^{13}C NMR (125 MHz, $CDCl_3$) δ, 155.44, 140.46, 140.24, 135.41, 129.92, 129.80, 128.62, 128.20, 127.69, 126.91, 126.73, 125.98, 125.56, 119.33, 117.69, 34.63.

EI m/z = 372.7, calculated for $C_{22}H_{16}N_2S_2$: 372.50

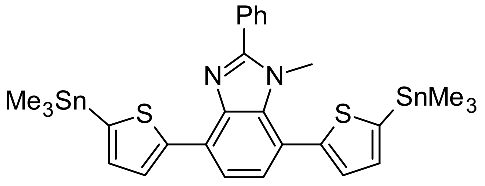

1-Methyl-2-phenyl-4,7-bis(5'-trimethyltinthiophene)benzimidazole, M1

A solution of 1-methyl-2-phenyl-4,7-bi(thiophene)benzimidazole, **2** (0.744 g, 2 mmol) in 10 ml of anhydrous THF was cooled to –78 °C under Ar. n-butyllithium (1.8 ml, 4.5 mmol, 2.5

M in hexanes) was added dropwise and the mixture was stirred at –78 °C for 20 min., then at RT for 2 h. The solution was cooled again to –78 °C and trimethyltin chloride (6 ml, 6 mmol, 1 M in hexanes) was added. The solution was allowed to warm to RT and stirred overnight. Then the reaction was quenched with 50 ml of NH_4Cl solution and extracted with diethyl ether. The organic phase was washed with water and dried over Na_2SO_4. The solvent was evaporated to give the product as a colorless oil (1.375 g, 1.97 mmol, 98% yield) which was used for the next step without further purification.

^{1}H NMR (500 MHz, $CDCl_3$) δ, 8.29 (d, J = 3.5 Hz, 1H), 7.84–7.82 (m, 2H), 7.58 (d, J = 7.5 Hz, 1H), 7.54–7.50 (m 3H), 7.27–7.25 (m, 3H), 7.20 (d, J = 3 Hz, 1H), 3.52 (s, 3H), 0.42 (s, 9H), 0.40 (s, 9H).

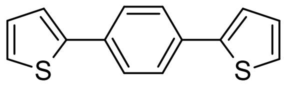

1,4-Di(thiophen-2-yl)benzene, 3

1,4-diiodobenzene (1.65 g, 5 mmol), thiophene-2-boronic acid pinacol ester (2.31 g, 11 mmol), BTENCl (0.06 g), tetrakis(triphenylphosphine) palladium(0) (144.5 mg, 0.125 mmol), toluene 15 ml and 15 ml of degassed 2 M water solution of Na_2CO_3 were added to the reaction flask under Ar. The mixture was stirred and heated at 100 °C for 24 h. After cooling the solution was poured into 50 ml of water and extracted with ethyl acetated. The organic phase was dried over $MgSO_4$. The crude product was purified by gel column chromatography eluting with dichloromethane : hexanes, 2:1 to give the product as a gold solid (0.92 g, 3.8 mmol) with 76% yield.

^{1}H NMR (500 MHz, $CDCl_3$) δ, 7.63 (s, 4H), 7.34 (dd, J = 3.6, 1.2 Hz, 2H), 7.29 (dd, J = 5.1, 1.2 Hz, 2H), 7.10 (dd, J = 5.1, 3.6 Hz, 2H).

^{13}C NMR (125 MHz, $CDCl_3$) δ, 151.75, 140.55, 135.67, 127.15, 126.75, 118.34

EI m/z = 242.6, calculated for $14_2H_{10}S_2$: 242.353

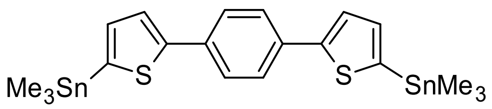

1,4-Di(5-trimethylstannylthiophen-2-yl)benzene, M2

A solution of 1,4-di(thiophen-2-yl)benzene, **3** (0.484 g, 2 mmol) in 10 ml of anhydrous THF was cooled to –78 °C under Ar. n-butyllithium (1.8 ml, 4.5 mmol, 2.5 M in hexanes) was added dropwise and the mixture was stirred at –78 °C for 20 min., then at RT for 2 h. The solution was cooled again to –78 °C and trimethyltin chloride (6 ml, 6 mmol, 1 M in hexanes) was added. The solution was allowed to warm to RT and stirred overnight. Then the reaction was quenched with 50 ml of NH_4Cl solution and extracted with diethyl ether. The organic phase was washed with water and dried over $MgSO_4$. The solvent was evaporated to give the product as a brown-yellow solid, which was recrystallized from isopropanol to obtain gold flakes (1.02 g, 1.8 mmol, 90% yield).

^{1}H NMR (500 MHz, $CDCl_3$) δ, 7.62 (s, 4H), 7.44 (d, J = 4.0 Hz, 2H), 7.18 (d, J = 4.0 Hz, 2H), 0.41 (s, 18H).

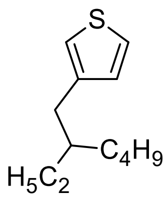

3-(2-ethylhexyl)thiophene, 4

^{1}H NMR (500 MHz, $CDCl_3$) δ, 7.22 (dd, J = 5.0, 3.0 Hz, 1H), 6.91–6.89 (m, 2H), 2.57 (d, J = 6.5 Hz, 2H), 1.58–1.54 (m, 1H), 1.32–1.23 (m, 8 H), 0.91–0.85 (m, 6H).

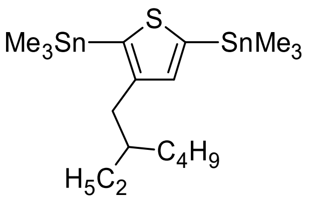

4-(2-ethylhexyl)thiophen-2-yl)trimethylstannane, 5

^{1}H NMR (500 MHz, $CDCl_3$) δ, 7.17 (d, J = 1.0 Hz, 1H), 6.97 (d, J = 1.0 Hz, 1H), 2.59 (d, J = 6.5 Hz, 2H), 1.58–1.56 (m, 1H), 1.32–1.20 (m, 8H), 0.91–0.85 (m, 6H), 0.35 (s, 9H).

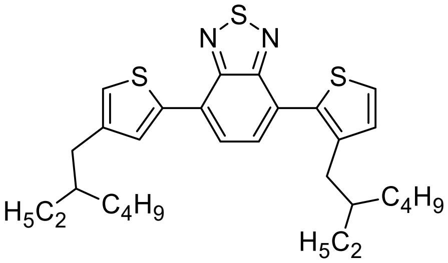

4,7-Bis(4-(2-ethylhexyl)thiophen-2-yl)benzo[1,2,5]thiadiazole, 6

^{1}H NMR (500 MHz, $CDCl_3$) δ, 7.94 (d, J = 1.5 Hz, 2H), 7.83 (s, 2H), 7.02 (d, J = 1.5 Hz, 2H), 2.63 (d, J = 7.0 Hz, 4H), 1.68–1.65 (m, 2H), 1.37–1.29 (m, 16H), 0.92–0.89 (m, 12H).

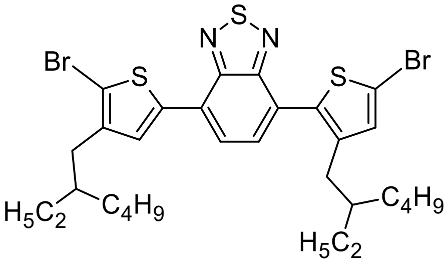

4,7-Bis(5-bromo-4-(2-ethylhexyl)thiophen-2-yl)benzo[1,2,5]thiadiazole, M3

^{1}H NMR (500 MHz, $CDCl_3$) δ, 7.75 (m, 4H), 2.58 (d, J = 7.0 Hz, 4H), 1.77–1.70 (m, 2H), 1.39–1.30 (m, 16H), 0.91–0.88 (m, 12H).

2,5-Bis(2-octyldodecyl)3,6-di(thiophen-2-yl)[3,4-c]pyrrole-1,4(2H,5H)-dione, 7

^{1}H NMR (500 MHz, $CDCl_3$) δ, 8.87 (dd, J = 4.0, 1.0 Hz, 2H), 7.61 (dd, J = 5.0, 1.0 Hz, 2H), 7.27–7.25 (m, 2H), 4.02 (d, J = 8.0 Hz, 4H), 1.94–1.88 (m, 2H), 1.30–1.20 (m, 64H), 0.88-0.84 (m, 12H).

3,6-Bis(5-bromothiophen-2-yl)-2,5-bis(2-octyldodecyl)pyrrolo[3,4-c]pyrrole-1,4(2H,5H)-dione, M4

^{1}H NMR (500 MHz, $CDCl_3$) δ, 8.62 (d, J = 4.5 Hz, 2H), 7.23 (d, J = 4.5 Hz, 2H), 3.92 (d, J = 8.0 Hz, 4H), 1.92–1.88 (m, 2H), 1.30–1.20 (m, 64H), 0.88–0.84 (m, 12H).

General method of Still coupling polymerization

In a glove box, bis(trimethylstannyl) derivative (0.2 mmol), dibromoderivative (0.2 mmol) were added to a microwave tube and the flask was purged several times with Ar. $Pd_2(dba)_3$ (0.01 mmol) and P(*o*-tol)$_3$ (0.04 mmol) were then added and the mixture was dissolved in 4 ml of chlorobenzene. The tube was sealed with a Teflon cap. The reaction mixture was heated to 100 °C for 1 min., to 135 °C for 1 min. and to 160 °C for 1 h in the microwave. After cooling tributyl(thiophene-2-yl)stannane (0.1ml) was added and the tube was heated to 160 °C for 10 min., followed by the addition of 2-bromothiophene (0.5 ml) and heating to 160 °C for 10 min. The crude polymer was precipitated in methanol. The solid was washed with methanol, acetone and hexanes. The main fraction was dissolved in chloroform, precipitated in methanol and vacuum dried.

Polymer P1

1-Methyl-2-phenyl-4,7-bis(5'-trimethyltinthiophene)benzimidazole, **M1** (140 mg, 0.2 mmol), 4,7-bis(5-bromo-4-(2-ethylhexyl)thiophen-2-yl)benzo[1,2,5]thiadiazole, **M3** (136.4 mg, 0.2 mmol) and $Pd_2(dba)_3$ (9.16 mg, 0.01 mmol) and P(*o*-tol)$_3$ (12.2 mg, 0.04 mmol) in 4 ml of chlorobenzene were used for the polymerization. The coupling reaction was performed as described above. The product was obtain as a dark red solid (150 mg). M_n = 5.5 kDa, M_w = 8.4 kDa, PDI = 1.53 (due to the limitation of GPC system only polymer fractions soluble in dichloromethane have been determined).

^{1}H NMR (500 MHz, $CDCl_3$) δ, 8.26 (1H), 7.98 (2H), 7.86 (4H), 7.61 (1H), 7.54 (3H), 7.32 (3H), 7.13 (1H), 3.67 (3H), 2.88 (4H), 1.84 (2H), 1.54–1.30 (16 H), 0.90 (12H).

IR (cm^{-1}) 3062, 2953, 2923, 2855, 1577, 1485, 1434, 1330, 1203, 1064, 862, 798, 776, 700.

Anal. Calculated for $C_{52}H_{52}N_4S_5$: C, 69.91; H, 5.87; N, 6.27; S, 17.95.

Anal. Found: C, 69.52; H, 5.77; N, 6.18; S, 17.63.

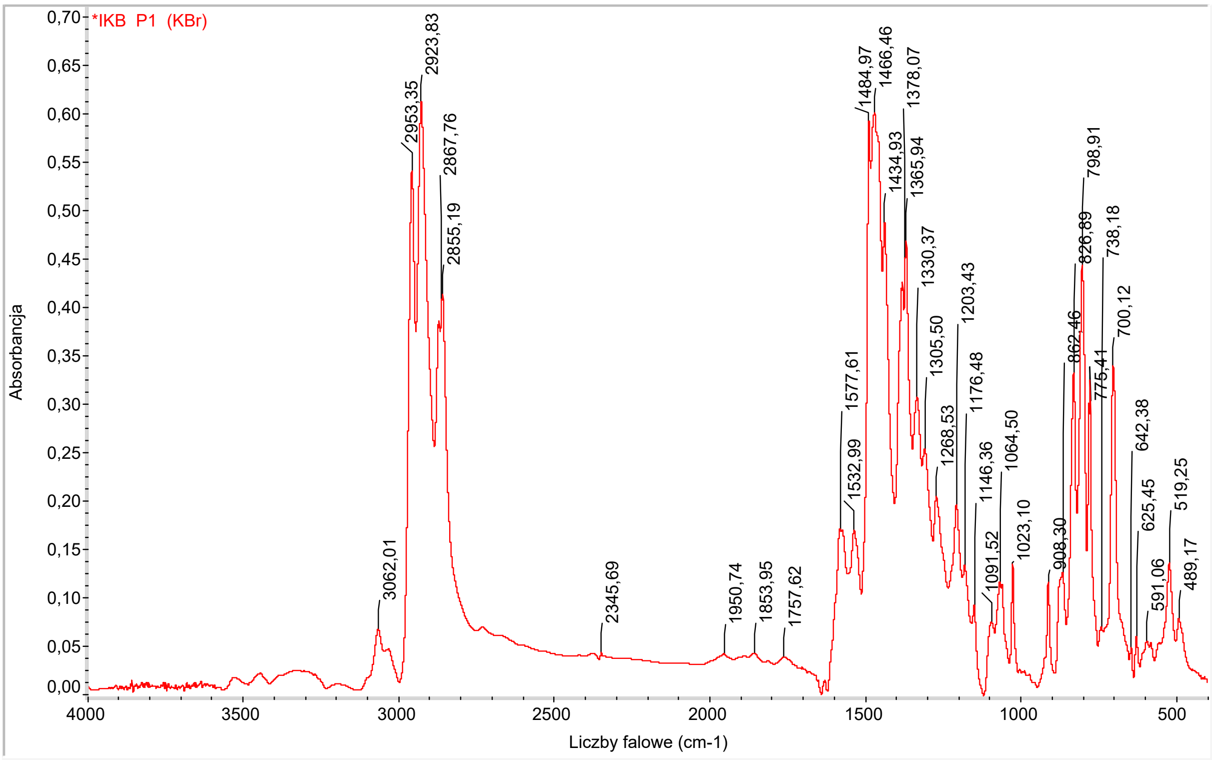

Figure S1. FTIR spectrum of the polymer **P1**.

Polymer P2

1-Methyl-2-phenyl-4,7-bis(5'-trimethyltinthiophene)benzimidazole, **M1** (140 mg, 0.2 mmol), 4,7-bis(5-bromo-4-(2-ethylhexyl)thiophen-2-yl)benzo[1,2,5]thiadiazole, **M3** (68.2 mg, 0.1 mmol) and 3,6-bis(5-bromothiophen-2-yl)-2,5-bis(2-octyldodecyl)pyrrolo[3,4-c]pyrrole-1,4(2H,5H)-dione, **M4** (102 mg, 0.1 mmol) $Pd_2(dba)_3$ (9.16 mg, 0.01 mmol) and P(*o*-tol)$_3$ (12.2 mg, 0.04 mmol) in 4 ml of chlorobenzene were used for the polymerization. The coupling reaction was performed as described above. The product was obtain as a dark blue solid (190 mg). M_n = 5.5 kDa, M_w = 8.2 kDa, PDI = 1.49 (due to the limitation of GPC system only polymer fractions soluble in dichloromethane have been determined).

IR (cm^{-1}) 3065, 2952, 2923, 2852, 1662, 1552, 1466, 1453, 1367, 1225, 1067, 799, 775, 701.

Anal. Calculated for $C_{128}H_{152}N_8O_2S_9$: C, 72.41; H, 7.21; N, 5.28; O, 1.51' S, 13.59.

Anal. Found: C, 71.41; H, 7.22; N, 5.04; S, 12.42.

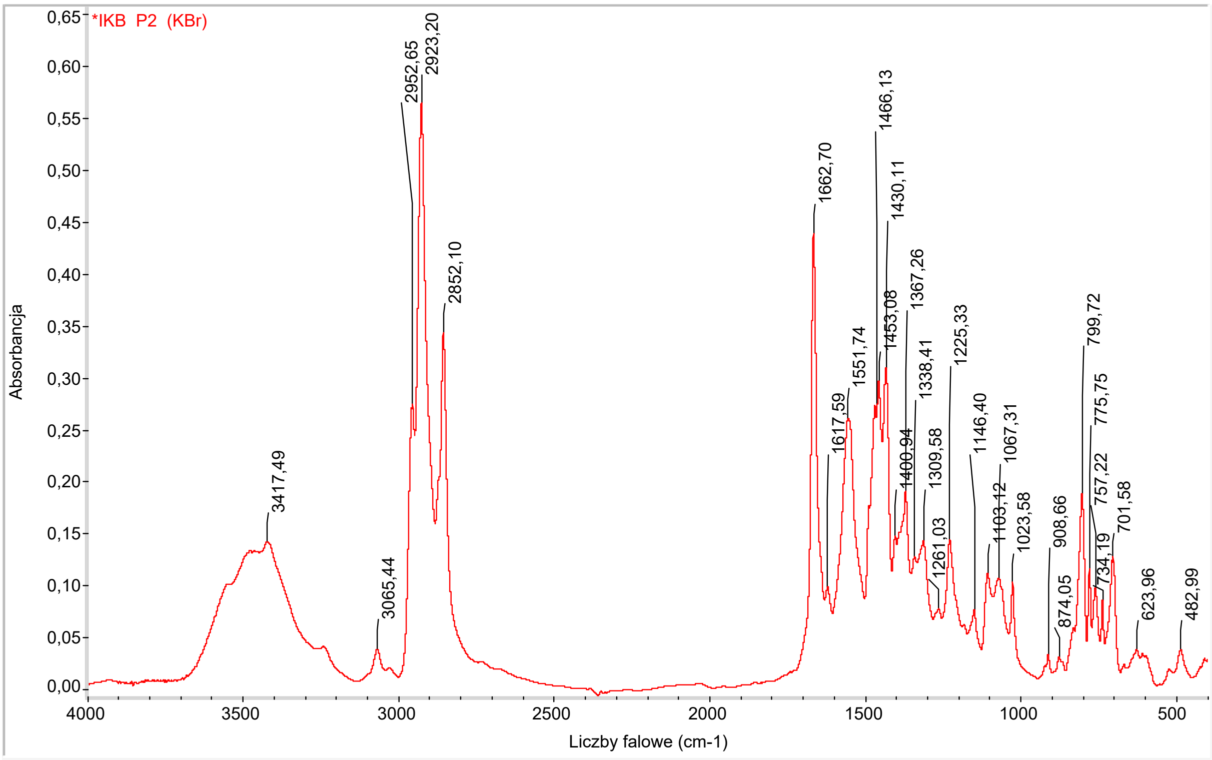

Figure S2. FTIR spectrum of the polymer **P2**.

Polymer P3

1,4-Di(5-trimethylstannylthiophen-2-yl)benzene, **M2**, (113.5 mg, 0.2 mmol) 4,7-bis(5-bromo-4-(2-ethylhexyl)thiophen-2-yl)benzo[1,2,5]thiadiazole, **M3** (68.2 mg, 0.1 mmol) and 3,6-bis(5-bromothiophen-2-yl)-2,5-bis(2-octyldodecyl)pyrrolo[3,4-c]pyrrole-1,4(2H,5H)-dione, **M4** (102 mg, 0.1 mmol), $Pd_2(dba)_3$ (9.16 mg, 0.01 mmol) and P(*o*-tol)$_3$ (12.2 mg, 0.04 mmol) in 4 ml of chlorobenzene were used for the polymerization. The coupling reaction was performed as described above. The product was obtain as a dark blue solid (170 mg). M_n = 6.6 kDa, M_w = 13.4 kDa, PDI = 2.01 (due to the limitation of GPC system only polymer fractions soluble in dichloromethane have been determined).

IR (cm^{-1}) 3066, 2953, 2921, 2851, 1661, 1542, 1486, 1429, 1224, 1068, 825, 788, 732.

Anal. Calculated for $C_{112}H_{140}N_4O_2S_9$: C, 72.20; H, 7.57; N, 3.02; O, 1.72; S, 15.49.

Anal. Found: C, 71.84; H, 7.23; N, 2.89; S, 15.17.

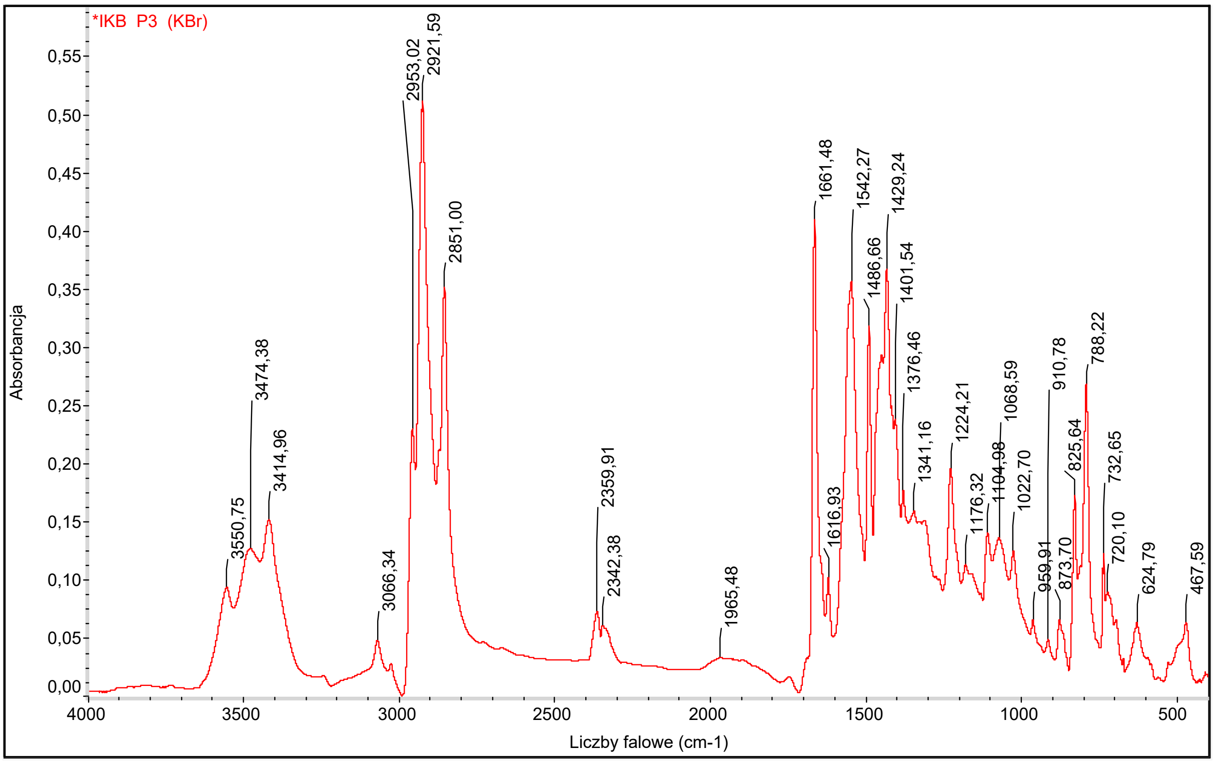

Figure S3. FTIR spectrum of the polymer **P3**.

Polymer P4

1-Methyl-2-phenyl-4,7-bis(5'-trimethyltinthiophene)benzimidazole, **M1** (140 mg, 0.2 mmol) and 3,6-bis(5-bromothiophen-2-yl)-2,5-bis(2-octyldodecyl)pyrrolo[3,4-c]pyrrole-1,4(2H,5H)-dione, **M4** (204 mg, 0.2 mmol), $Pd_2(dba)_3$ (9.16 mg, 0.01 mmol) and P(*o*-tol)$_3$ (12.2 mg, 0.04 mmol) in 4 ml of chlorobenzene were used for the polymerization. The coupling reaction was performed as described above. The product was obtain as a dark blue solid (115 mg). M_n = 10.6 kDa, M_w = 20.8 kDa, PDI = 1.96 (due to the limitation of GPC system only polymer fractions soluble in dichloromethane have been determined).

IR (cm^{-1}) 3066, 2923, 2852, 1662, 1548, 1429, 1312, 1226, 1073, 799, 758, 702.

Anal. Calculated for $C_{76}H_{100}N_4O_2S_4$: C, 74.22; H, 8.19; N, 4.56; O, 2.60; S, 10.43.

Anal. Found: C, 73.93; H, 8.07; N, 4.26; S, 9.41.

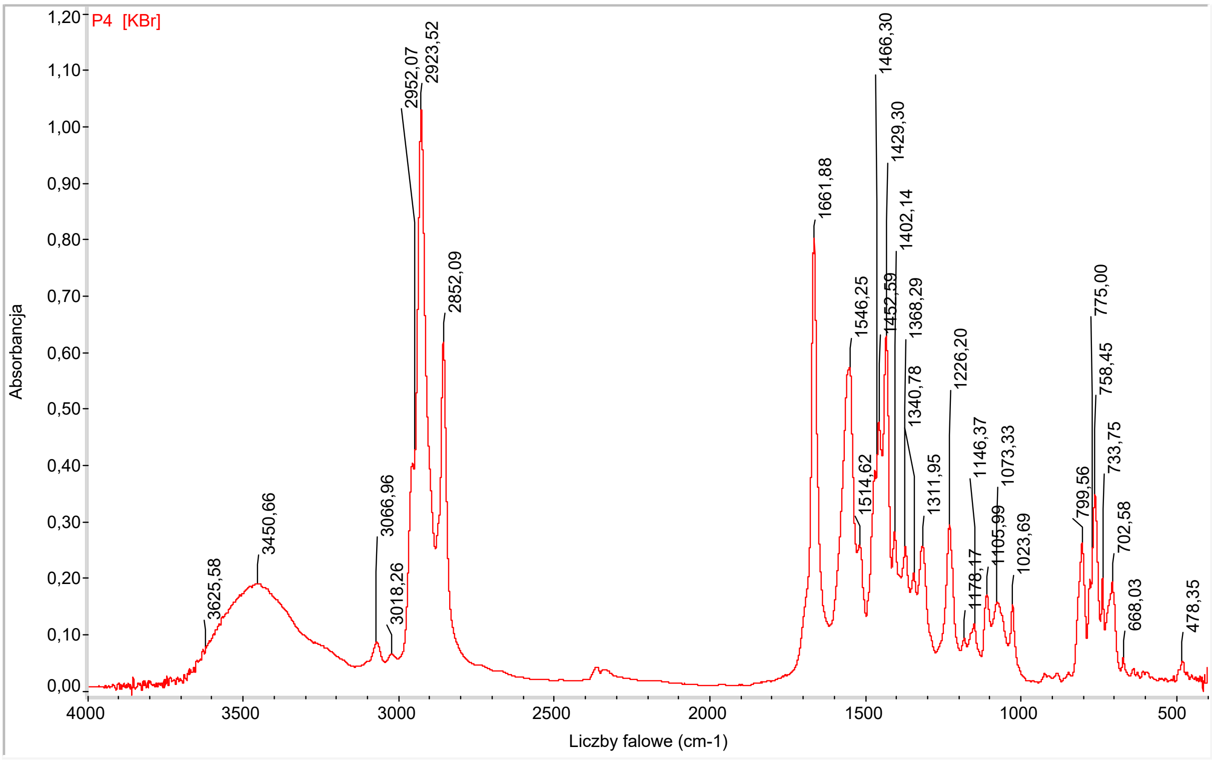

Figure S4. FTIR spectrum of the polymer **P4**.

Polymer P5

1,4-Di(5-trimethylstannylthiophen-2-yl)benzene, **M2**, (113.5 mg, 0.2 mmol) and 3,6-bis(5-bromothiophen-2-yl)-2,5-bis(2-octyldodecyl)pyrrolo[3,4-c]pyrrole-1,4(2H,5H)-dione, **M4** (204 mg, 0.2 mmol) $Pd_2(dba)_3$ (9.16 mg, 0.01 mmol) and P(*o*-tol)$_3$ (12.2 mg, 0.04 mmol) in 4 ml of chlorobenzene were used for the polymerization. The coupling reaction was performed as described above. The product was obtain as a dark blue solid (180 mg). M_n = 7.9 kDa, M_w = 10.9 kDa, PDI = 1.38 (due to the limitation of GPC system only polymer fractions soluble in dichloromethane have been determined).

IR (cm^{-1}) 3068, 2922, 2852, 1664, 1544, 1431, 1226, 1107, 787, 733.

Anal. Calculated for $C_{68}H_{94}N_2O_2S_4$: C, 74.27; H, 8.61; N, 2.55; O, 2.91; S, 11.66.

Anal. Found: C, 72.88; H, 8.16; N, 2.45; S, 11.26.

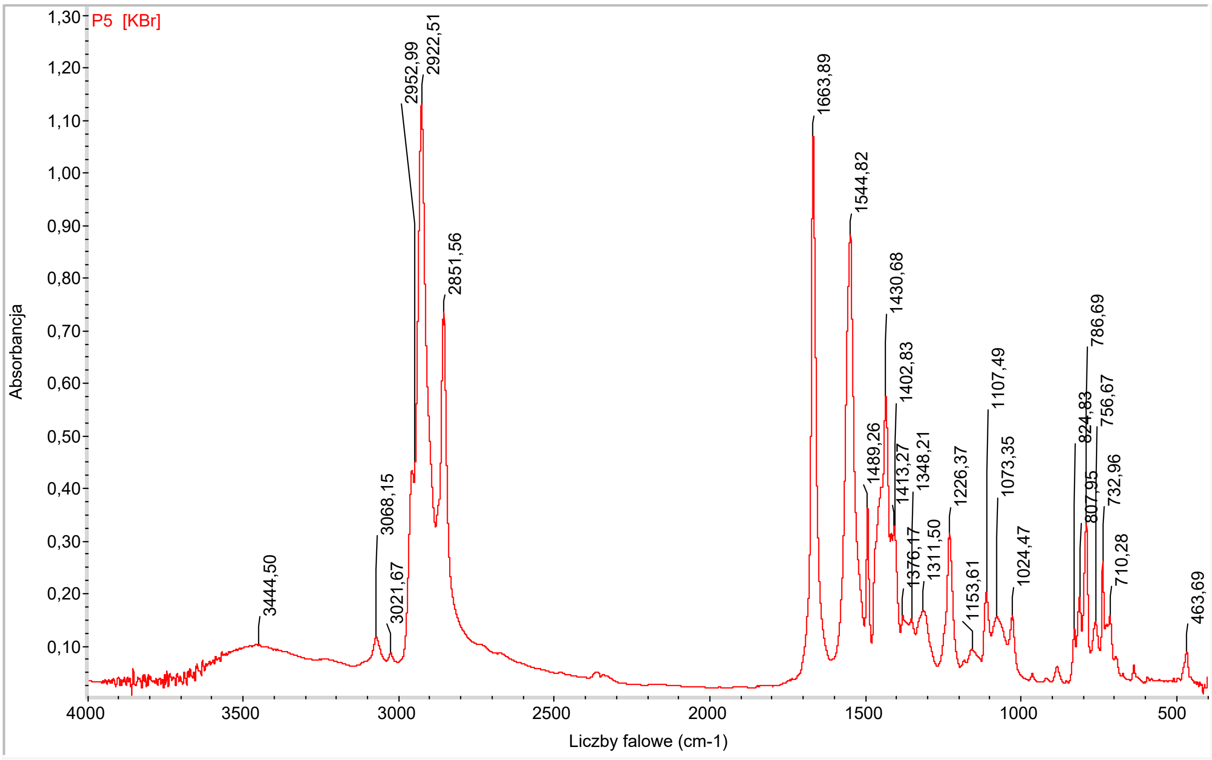

Figure S5. FTIR spectrum of the polymer **P5**.

Optical studies

The polymers were dissolved in chloroform or chlorobenzene and corresponding spectra in solutions or in solid-state films were registered using Carry 5000 (Varian) spectrometer.

The thin layers of polymers and the corresponding blends with $PC_{71}BM$ (in 1:2 molar ratio) were deposited on quartz substrates *via* drop casting and dried under vacuum. The emission spectra were registered using Edinburgh FS5 spectrofluorometer equipped with an enhanced range photomultiplier detector (PMT-EXT) applying front face geometry. The samples were excited at wavelengths corresponding to maxima of lowest energy absorption bands of polymer in chloroform solutions.

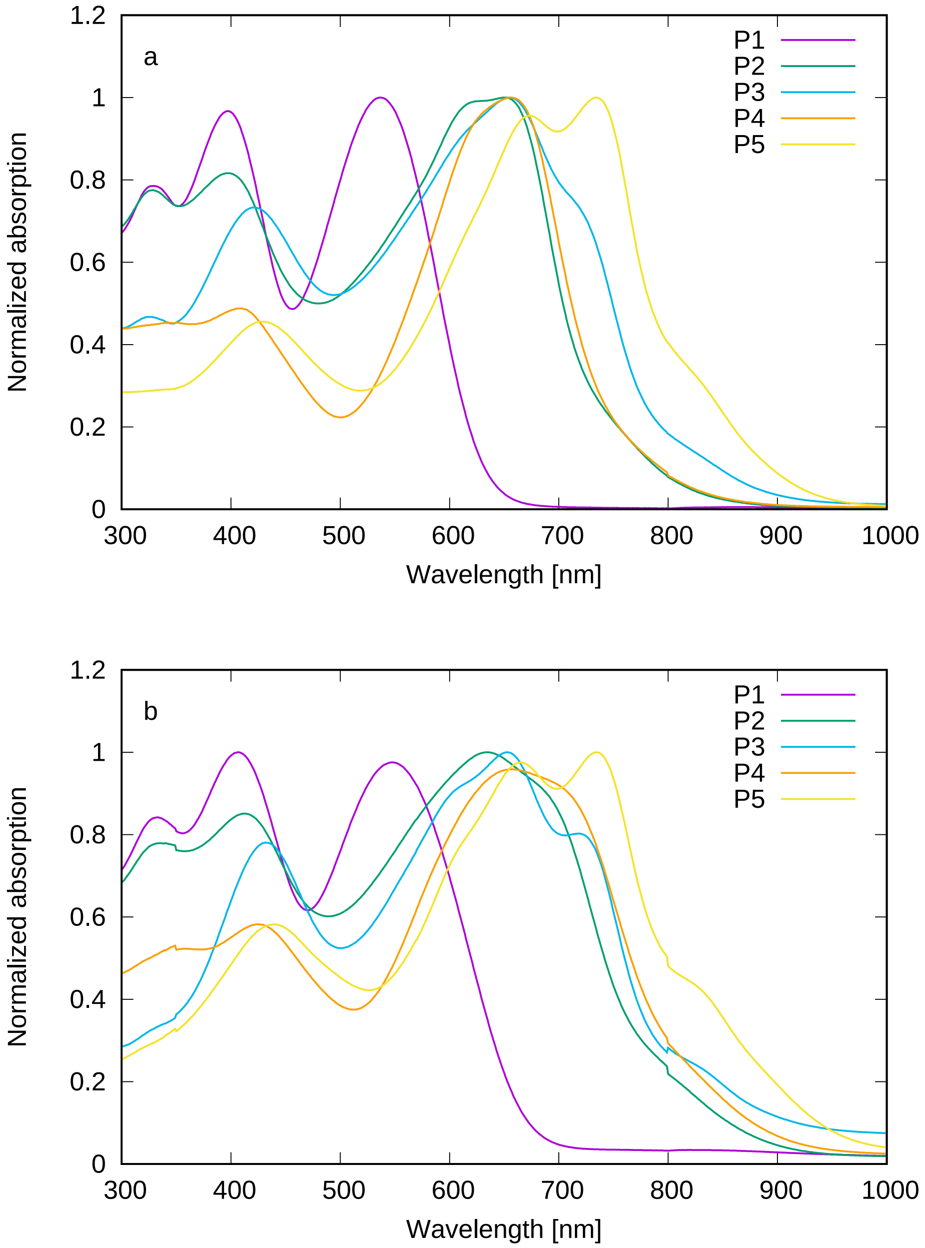

Figure S6. Absorption spectra of the polymers **P1**–**P5**: (a) in chlorobenzene solutions, (b) in solid-state films.

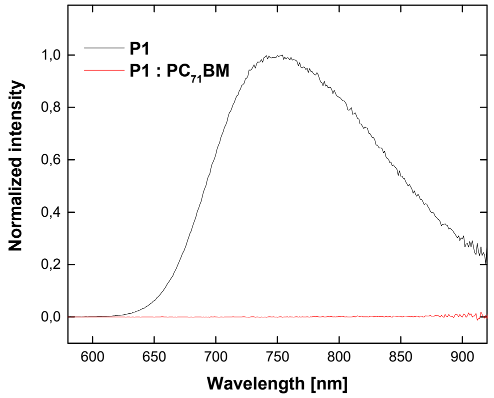

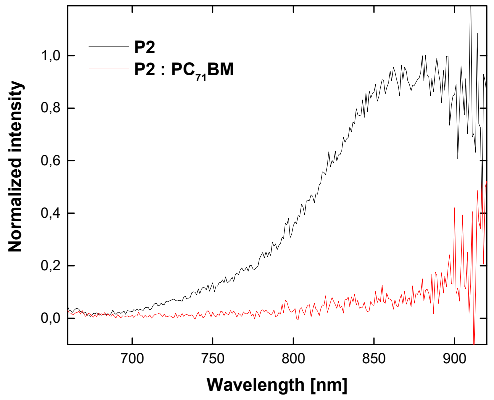

Figure S7. Photoluminescence spectra of neat films of **P1** and **P2** and their blends with $PC_{71}BM$ (1:2).

Electrochemical studies

Cyclic voltammograms, CV (scan rate 50 mV/s) and differential pulse voltammograms, DPV (modulation time: 50 ms, modulation amplitude: 10 mV, step potential: 5 mV) of the synthesized polymers dissolved in 0.1 M Bu_4NBF_4/dichloromethane electrolyte were registered using an Autolab potentiostat (EcoChemie, the Netherlands). The measurements were performed in an inert atmosphere, using a platinum disk working electrode of the surface area of 2 mm^2, a platinum wire counter electrode and an Ag/0.1 M Ag^+/acetonitrile reference electrode, whose potential was verified using the ferrocene couple at the end of each set of experiment.

Morphological studies – AFM measurements

The polymer thin films for microscopic investigations (of a thickness from monolayer to several molecular layers) were prepared by drop-casting from a solution of each investigated macromolecule in chlorobenzene or chloroform (~20–100 mg/L depending on the film thickness) on a freshly cleaved surface of mica (muscovite mica, SPI Supplies, USA). After evaporation the solvent in air, the samples were imaged in ambient conditions by means of atomic force microscopy. The AFM system Dimension Icon (Bruker) was applied. All images were collected in Tapping Mode. Standard AFM probes from Bruker were used. For each sample, microscopic investigations were repeated several times at different surface areas to get statistical information.

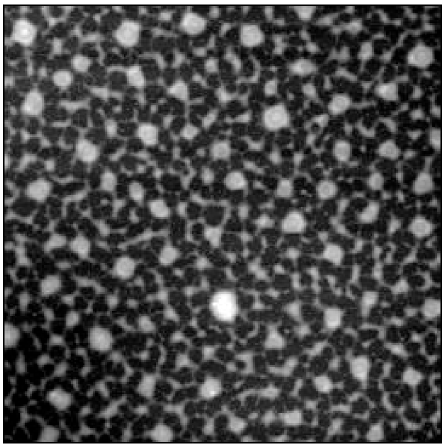

Figure S8 AFM images of **P2** layers deposited on mica from solution in chlorobenzene.

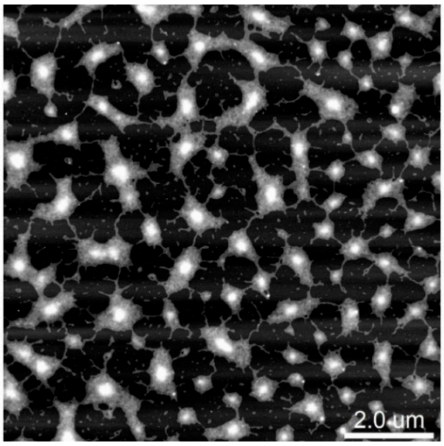

Figure S9. AFM images of **P4** layers deposited on mica from solution in chlorobenzene.

Device fabrication and characterization

Solar cells of architectures: ITO / PEDOT : PSS / polimer : $PC_{70}BM$ / InAl were fabricated on the glass substrates covered with conductive indium-tin oxide (ITO) forming 8 separated pixels with an area of 4 mm^2 each. First PEDOT : PSS layers of a thickness 40 nm were spincoated on ITO glass substrate with 5000 rpm for 60 s using an aqueous solution, and then the samples were annealed at 150 °C for 15 min in air. Then active layer solution was spincoated in an Ar glovebox with 900 rpm for 60 s. Deposited polymer-fullerene bulk heterojunctions (BHJ) layers of 90 nm thickness were dried in vacuum in glove box antechamber for 15 min in order to remove residual solvent. Finally, the InAl electrode of thickness 100 nm was deposited by thermal evaporation in vacuum (about 10^{-5} mbar). Total concentration of PTB7 : $PC_{70}BM$ solution was 25 mg/ml with 2:3 weight ratio.

A photovoltaic cell is seen in **Figure S10** It has 8 active pixels (brown rectangles) with metal contacts (white horizontal bars).

Current-voltage characteristics were measured using of Keithley 2450 Source Meter with Kickstart PC software. As a source of illumination a Newport VeraSol-2 LED Class AAA Solar Simulator of 1000 W/m^2 power output and AM1.5G spectrum was applied.

Photocurrent spectroscopy was measured in the range from 0.7 eV to 4 eV using the light of the incandescent lamp passing through the monochromator. The current was measured in a short circuit mode (I_{SC}) with a Keithley picoammeter. For external quantum efficiency (EQE) calculation, the light intensity spectrum was measured with PM320 power-meter.

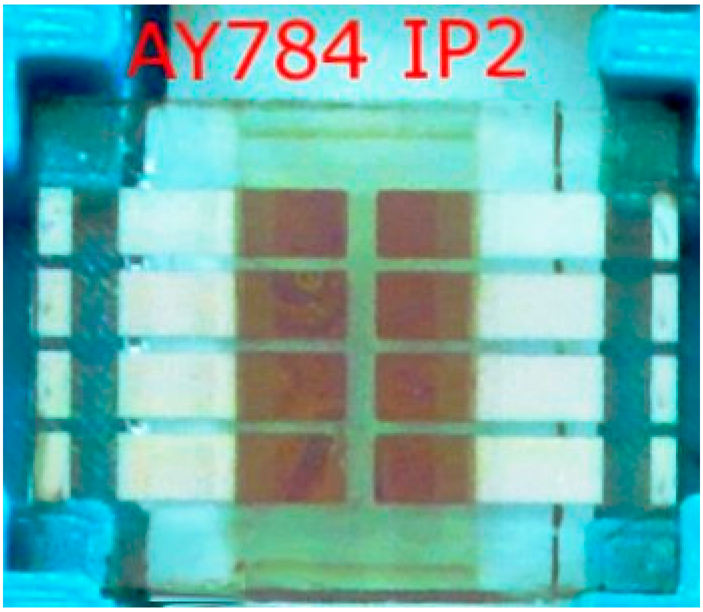

Figure S10. Photograph of solar cell with **P2**:PCBM heterojunction.

Table S1. Photoelectric parameters of the investigated solar cells.

cell type	V_{oc} [V]	J_{SC} [mA/cm^2]	FF [%]	Rs [Ω*cm^2]	Rsh [Ω*cm^2]	PCE [%]	3 cells PCE$_{av.}$ [%]	8 cells PCE$_{av.}$ [%]
**P2**:PC70BM (CB+3%DIO)	0.422	2.20	30.1	7.41	249	0.279	0.277	0.229
**P2**:PC70BM (CF+6%DCB)	0.499	1.46	27.4	8.62	376	0.199	0.187	0.142
**P4**:PC70BM (CB+3%DIO)	0.531	1.94	39.6	4.21	589	0.408	0.381	0.335
**P4**:PC70BM (CF+6%DCB)	0.463	4.45	30.0	2.92	141	0.619	0.603	0.479

CF - chloroform; CB - chlorobenzene, DCB - o-dichlorobenzene; DIO - diiodobenzene

Morphology of the active layer

Morphology of the active layer was investigated with optical microscope using Nomarsky contrast.

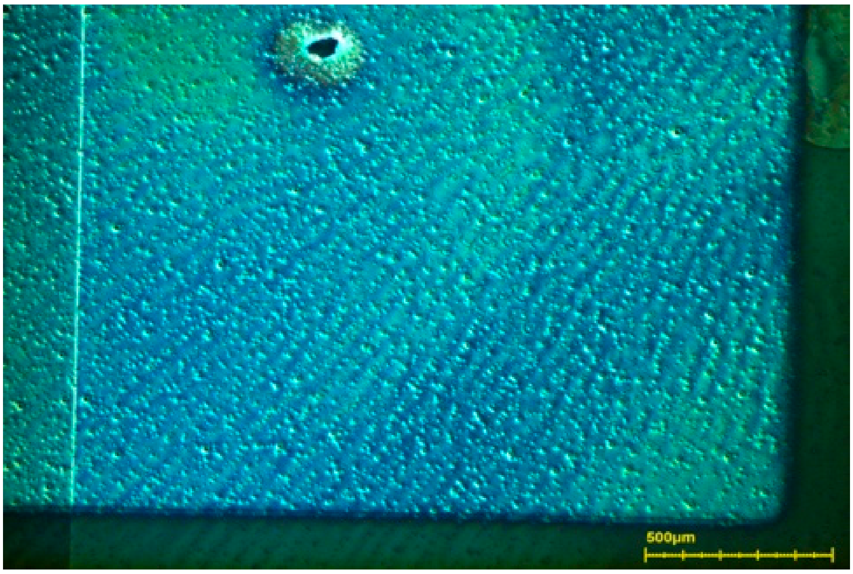

Figure S11. Solar cell **P2**:$PC_{70}BM$ – a lot of small bubbles are visible caused by evaporation of the solvent. The bubbles reduce the working area.

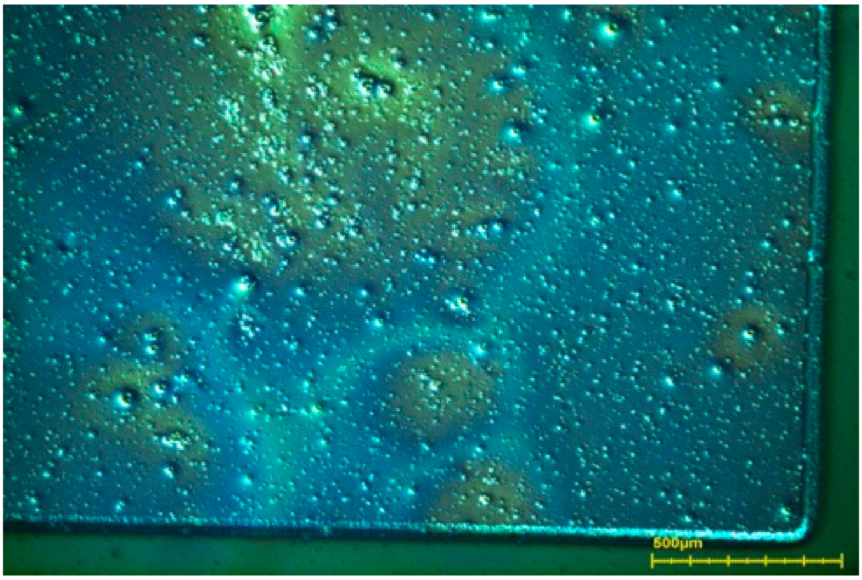

Figure S12. Solar cell **P4**:$PC_{70}BM$ – The bubbles are bigger than in the case of P2 but the flat area between them is greater than in the case of P2. This can be a reason of the higher efficiency observed for P4.

Bibligraphy

1. Wrobel, Z.; Stachowska, K.; Grudzien, K.; Kwast, A. N-Aryl-Nitrosoanilines as Intermediates in the Two-step Synthesis of Substituted 1,2-Diarylbenzimidazoles from Simple Nitroarenes. *Synlett.*, **2011**, *10*, 1439–1443.

2. Conboy, G.; Taylor, R.G.D.; Findlay, N.J.; Kanibolotsky, A.L.; Inigo, A.R.; Ghosh, S.S.; Ebenhoch, B.; Jagadamma, L.K.; Thalluri, G.K.V.V.; Sajjad, M.T.; Samuel, I.D.W.; Skabara, P.J. Novel 4,8-Benzobisthiazole Copolymers and Their Field-Effect Transistor and Photovoltaic Applications. *J. Mater. Chem. C*, **2017**, *5*, 11927–11936.
